# A Novel R2R3-MYB Transcription Factor BpMYB106 of Birch (*Betula platyphylla*) Confers Increased Photosynthesis and Growth Rate through Up-regulating Photosynthetic Gene Expression

**DOI:** 10.3389/fpls.2016.00315

**Published:** 2016-03-22

**Authors:** Chenguang Zhou, Chenghao Li

**Affiliations:** State Key Laboratory of Tree Genetics and Breeding, Northeast Forestry UniversityHarbin, China

**Keywords:** *Betula platyphylla*, BpMYB106, photosynthesis, growth rate, trichome, protein-DNA interaction

## Abstract

We isolated a R2R3-MYB transcription factor BpMYB106, which regulates photosynthesis in birch (*Betula platyphylla* Suk.). *BpMYB106* mainly expresses in the leaf and shoot tip of birch, and its protein is localized in the nucleus. We further fused isolated a 1588 bp promoter of BpMYB106 and analyzed it by PLACE, which showed some *cis*-acting elements related to photosynthesis. BpMYB106 promoter β-glucuronidase (GUS) reporter fusion studies gene, the result, showed the GUS reporter gene in transgenic birch with BpMYB106 promoter showed strong activities in shoot tip, cotyledon margins, and mature leaf trichomes. The overexpression of *BpMYB106* in birch resulted in significantly increased trichome density, net photosynthetic rate, and growth rate as compared with the wild-type birch. RNA-Seq profiling revealed the upregulation of several photosynthesis-related genes in the photosynthesis and oxidative phosphorylation pathways in the leaves of transgenic plants. Yeast one-hybrid analysis, coupled with transient assay in tobacco, revealed that BpMYB106 binds a MYB binding site MYB2 in differentially expressed gene promoters. Thus, BpMYB106 may directly activate the expression of a range of photosynthesis related genes through interacting with the MYB2 element in their promoters. Our study demonstrating the overexpression of BpMYB106—a R2R3-MYB transcription factor—upregulates the genes of the photosynthesis and oxidative phosphorylation pathways to improve photosynthesis.

## Introduction

Photosynthesis in green plants and other photosynthetic organisms provides a constant source of energy to fuel life on earth by converting light into biochemical energy (Singh et al., 2014). As the growth rate of plants directly reflects the photosynthetic rate (Evans, 2013), enhancing the photosynthetic capacity of plants is a promising approach to increase agriculture and forestry productivity. Conventional breeding strategies for photosynthesis improvement have long cycles and yield uncertain results (Richards, 2000). Genetic engineering has short cycles and directionality and is a better approach to enhance plant photosynthesis. Many protein-coding genes were encoded in nucleus and chloroplasts function together to assist the photosynthetic process (Berry et al., 2013). Transcription factors (TFs) are proteins with a DNA domain that binds to the *cis*-acting elements present in the promoter of target genes (Saibo et al., 2009). TFs can regulate the expression of a set of genes (Mitsuda and Ohme-Takagi, 2009) to promote photosynthetic gene activity, which in turn improves the plant photosynthetic efficiency. Ambavaram et al. (2014) showed that the expression of a TF, HYR enhances photosynthetic efficiency of rice under multiple environmental conditions. Besides, HYR, only a few TFs were identified as positive factors that can enhance photosynthetic capacity by activation of photosynthesis gene expression, especially in forest trees (Hymus et al., 2013; Kobayashi et al., 2013).

MYB transcription factors, which belong to one of the largest plant TF families, play crucial roles in plant growth and development (Zhao and Bartley, 2014). MYB proteins are classified based on the number of adjacent repeats with R1, R2R3, 3R, and 4R proteins having one, two, three, and four repeats, respectively (Zhao and Bartley, 2014). R2R3-MYB proteins are the most abundant of the MYB classes in plants (Dubos et al., 2010). MYB TFs that regulate photosynthetic genes without stress conditions were cloned and characterized from different plant species such as CCA1 (CIRCADIAN CLOCK ASSOCIATED 1) from *Arabidopsis* (Wang et al., 1997; Wang and Tobin, 1998), and HvMCB1 and HvMCB2 from barley (Churin et al., 2003). However, most MYB genes belong to the R1/2-MYB subfamily members, and there are no reports on R2R3-MYB TFs that regulate photosynthesis-related genes directly.

Trichomes are specialized structures that develop from epidermal cells in the aerial parts of plants (An et al., 2011). An increase in trichome density is correlated to decreased transpiration and photosynthesis, which negatively impact plant productivity (Ehleringer et al., 1976; Pérez-Estrada et al., 2000). Plett et al. (2010) showed that the increased expression of the gene *PtrMYB186* significantly increased trichome density in fuzzy mutants lines obtained from *Populus tremula* × *Populus alba 717-1B4*. The mutant lines showed increased foliar trichome density, photosynthetic capacity, transpiration, growth rate, and pest resistance (Plett et al., 2010). This study showed that there may be a vast set of unexplored aspects of plant development that indirectly affect photosynthesis and plant growth. *AtMYB106* of *Arabidopsis thaliana* and *PtrMYB186* of *Populus trichocarpa*, which belong to *Ptr*R2R3-MYB clade 15 of R2R3-MYB subfamily (Wilkins et al., 2009), are homologous genes that function in trichome development (Jakoby et al., 2008). AtMYB106 is a negative regulator of trichome branching and does not affect trichome density (Jakoby et al., 2008). In contrast, PtrMYB186 is a positive regulator of trichome density but does not affect trichome branching in poplar (Plett et al., 2010). Although the same class of TFs regulated the trichome density in different species, the plant phenotypes were significantly different.

Birch (*Betula platyphylla* Suk.) is an important forest species widely distributed in northeastern China, and has many applications in architecture, furniture, and paper production (Liu et al., 2012). In the present study, we cloned BpMYB106—a R2R3-MYB transcription factor that regulates photosynthesis. We used Agro-BpMYB106 constructs to transform birch and obtain *BpMYB106* overexpression lines. We conducted the molecular and physical analyses involved in trichome density, photosynthetic capacity, and growth rate in transgenic birch. Gene expression profile studies proved the function of BpMYB106 in birch photosynthesis. Our results showed that *BpMYB106* gene overexpression improved photosynthesis and growth rate in birch via up regulating genes of photosynthesis and oxidative phosphorylation.

## Materials and methods

### Plant materials, vector construction, and plant transformation

Experimental studies were carried out on birch (*B. platyphylla* Suk.) that was cultivated under natural conditions in a field at Northeast Forestry University in Harbin, China. The complete *BpMYB106* reading frame was isolated from *B. platyphylla* and cloned in pROKII at the *BamHI-SacI* sites to obtain the pROKII-*BpMYB106* construct. *BpMYB106* was driven by a CaMV 35S (cauliflower mosaic virus 35S) promoter in the construct. The pROKII-*BpMYB106* construct was electroporated into *Agrobacterium tumefaciens* EHA105 and then introduced into birch as described by Huang et al. (2014). *BpMYB106* transgenic birch plants were selected in WPM medium (Lloyd and McCown, 1980) containing 30 mg/l kanamycin (Roche, Germany). Independent transformants, which have taken roots for 2 months were transferred to soil for hardening and plants grown in a glasshouse maintained at 25°C. All primers used for the cloning of cDNAs or plasmid constructions are listed in Table S6

### RNA isolation, cDNA preparation, and real-time quantitative RT-PCR (qRT-PCR)

RNA was isolated from the shoot, leaf, stem and root tissues by CTAB method (Jaakola et al., 2001). All of the qRT-PCR reactions were carried out on RQ1 RNase-free DNase (Promega, Madison, WI, USA). cDNA synthesis from total RNA was performed using the PrimeScript™ RT reagents Kit (Takara, Otsu, Japan) according to the manufacturer's instructions. For real-time (RT) PCR analysis, assays were performed using SYBR® Premix Ex Taq™ II (Takara) kit. PCR was performed on an MJ Research OpticonTM^2^ instrument (Bio-Rad, Hercules, CA, USA). Primers were designed as 17–25 bp oligomers with Tm 60–65 to amplify a product 80–150 bp long. Quantification was performed by 2^Δ*ΔC*(*t*)^ using *18s* as housekeeping gene for normalization. Primers used for the quantitative RT-PCR are listed in Table S7.

### Cloning and analysis of *BpMYB106* promoter and transient transformation

The promoter of BpMYB106 was cloned by genome walking method according to the Genome Walking kit (Takara). The *cis*-acting elements of the promoter sequence were predicted by PLACE online database (http://www.dna.affrc.go.jp/PLACE/).

A 1500 bp-long sequence that is prior to the predicted transcription start site “ATG,” was ligated with *HindIII* and *XbaI* and cloned into pBI121-GUS vector instead of CaMV35S promoter to construct the GUS fusion vector pBpMYB106::GUS. *Agrobacterium*-mediated birch transient transformation and histochemical staining were performed as described (Zheng et al., 2012).

### Subcellular localization of *BpMYB106* protein

The whole coding sequence of *BpMYB106*, with removed stop code, was ligated with *SalI* and *SpeI*-digested pBI121 vector to generate pBI121-*BpMYB106-GFP* containing *BpMYB106-GFP* fusion construct under the control of CaMV 35S promoter. The construct was confirmed by sequencing and used for transient transformation of onion (*Allium cepa*) epidermis via a PDS-1000/He System (Bio-Rad, Hercules, CA, USA). After 24 h of incubation, GFP fluorescence in transformed onion cells was observed under a confocal laser microscope (Zeiss LSM700, Jena, Germany).

### Physiological studies

For each experiment, 15—20 plants per independent transgenic line were grown under natural light in pots as described. Measurable leaves were the fourth or fifth leaf from the top in 45-day-old plants. The trichome phenotype leaf pubescence was analyzed using SEM (Nikon JCM-5000 NeoScope, Japan). Adaxial and abaxial side trichomes were counted from SEM images of fully expanded leaves (Plett et al., 2010).

Two separate growth trials were performed in early and mid-spring in 2014. The trees were watered every 3 days. Trees with similar growing status and height were chosen for growth comparison. The measurement period was 60 days, and measured height every 10 days.

Net photosynthetic rate (*A*), stomatal conductance (*Gs*), internal CO_2_ concentration (*Ci*), and transpiration rate (*E*) were measured using a LI6400 Portable Photosynthesis System (Li-Cor, Lincoln, NE). The measurements were taken between 10:00 and 11:30 a.m. under CO_2_ concentration of 400 μmol·mol^−1^ and PPFD (Photosynthetic Photon Flux Density) of 1400 mmol·m^−2^·s^−1^. Intrinsic water use efficiency was calculated as the *A/E* ratio. Flow rate was maintained at 200–250 mmol·s^−1^ so that the relative humidity inside the chamber was similar to the ambient condition.

For estimation of stomatal density, leaf epidermis (about 1 cm^2^) from the abaxial surface of fully expanded leaves (fourth leaf from top of 45-day-old plants) was peeled off with a pair of forceps and placed immediately in water and later mounted in 10% glycerol and observed under a light microscope (OLYMPUS B × 43, Tokyo, Japan). Stomata were counted in six different regions at the same position in three plants of each transgenic line and WT.

### RNA extraction, sequencing, and raw data processing

The fourth or fifth leaves from the top of birch were collected from WT and transgenic birch line 1 in 45-day-old plants, with same vigor in six plants per each line. Extracted RNA of six leaves from different plants per each line, respectively, mixed equally after concentration measurement.

For library construction, the total RNA samples were treated with DNase I to degrade any possible DNA contamination. RNA quality and quantity were determined by an Agilent 2100 bioanalyzer. Then the mRNA is enriched by using the oligo (dT) magnetic beads. Mixed with the fragmentation buffer, the mRNA was mixed with the fragmentation buffer to obtain short fragments (about 200 bp). First strand cDNA was synthesized using random hexamer-primer. Buffer, dNTPs, RNase H and DNA polymerase I are added to synthesize the second strand. The double strand cDNA was purified with magnetic beads. End repair and poly A addition were performed. Finally, sequencing adaptors are ligated to the fragments. The fragments are enriched by PCR amplification. During the QC step, Agilent 2100 Bioanaylzer and ABI StepOnePlus Real-Time PCR System are used to qualify and quantify of the sample library. The library products are ready for sequencing via Illumina HiSeq™ 2000 (Illumina, San Diego, CA, USA). Sequence data from this study were deposited in the NCBI Sequence Read Archive (SRA, http://www.ncbi.nlm.nih.gov/Traces/sra/) under the accession number SRP058150. Data filtering helped obtain high-quality reads as the clean reads (clean data). Clean reads were mapped to reference sequences and/or reference gene set using SOAPaligner/SOAP2 (Li et al., 2009). No more than two mismatches were allowed in the alignment.

### Identification and annotation of DEGs

For functional gene annotation, BLAST (-p blastx -e 1e-5 –m 7) sequences were annotated to Nr database of NCBI to GO terms using BLAST2GO (default parameters), and annotating to the KEGG database by BLAST (-p blastx -e 1e-5 –m 8). Gene differential expression analysis between WT and line 1 of birch (referring to “The significance of digital gene expression profiles,” Audic and Claverie, 1997) we used “FDR ≤ 0.001 and the absolute value of log2Ratio ≥1” as thresholds for determining the significance of gene expression difference. This analysis includes the screening of genes that are differentially expressed in samples, and GO functional enrichment analysis and KEGG pathway enrichment analysis for these DEGs.

We analyzed the DEGs of interest by bioinformatics. “Ultra Edit” tool was used to obtain DEGs promoter sequences, those 2000 bp-long sequences which prior to each transcription start site “ATG” *cis*-acting elements of each promoter sequence were predicted by PLACE online database.

### Yeast one-hybrid assay

Yeast one-hybrid assay was performed using the Yeastmaker™ Yeast transformation system 2 (Clontech, Palo Alto, CA, USA). The DNA fragment of three tandem copies of each element was synthesized and inserted directly into the multiple cloning sites of reporter plasmids of pHIS2. The whole coding sequence of *BpMYB106*, which removed terminated codes, was ligated into the *SmaI*-digested pGADT7-Rec2 vector to generate pGADT7-Rec2-*BpMYB106* vector. These two constructs were integrated into the genome of yeast strain Y187. The dual reporter strain was selected and maintained on synthetic dextrose (SD)/-Leu/-Trp (DDO), SD/-His/-Leu/-Trp (TDO), and TDO containing 30 mM 3-amino-1,2,4-triazole (3-AT) medium, respectively. The single yeast colony on TDO/30 mM 3-AT was activated to analyze the motifs recognized by BpMYB106 as described (Ji et al., 2014). All primers used for Y1H are listed in Table S8.

### *Agrobacterium*-mediated GUS transient assay in tobacco leaves

For construction of the reporter vectors, the minimal 35S (−46 to +10) promoter was amplified by PCR as described previously (Sun et al., 2010). pROKII-*BpMYB106* was used as the effector vector. Plasmids of reporter and effector vectors were introduced into the *Agrobacterium* tumefaciens strain EHA 105. The procedure of *Agrobacterium*-mediated transient assay was performed on the leaves of 6-week-old tobacco plants as described previously (Yang et al., 2000; Sun et al., 2010). All primers used for transient assay are listed in Table S9.

### Statistical analysis

The significance of correlations was tested by using linear regression, with *P* < 0.05 considered statistically significant. Means were compared by using two-tailed *t*-test and one-way ANOVA. All data analysis and plotting were performed with PASW Statistics 18.0 (SPSS Inc., Chicago, IL, USA) and Microsoft Office Excel (Microsoft, Redmond, WA, USA).

## Results

### Isolation, sequence analysis, and gene expression pattern of *BpMYB106*

**Figure 1:**
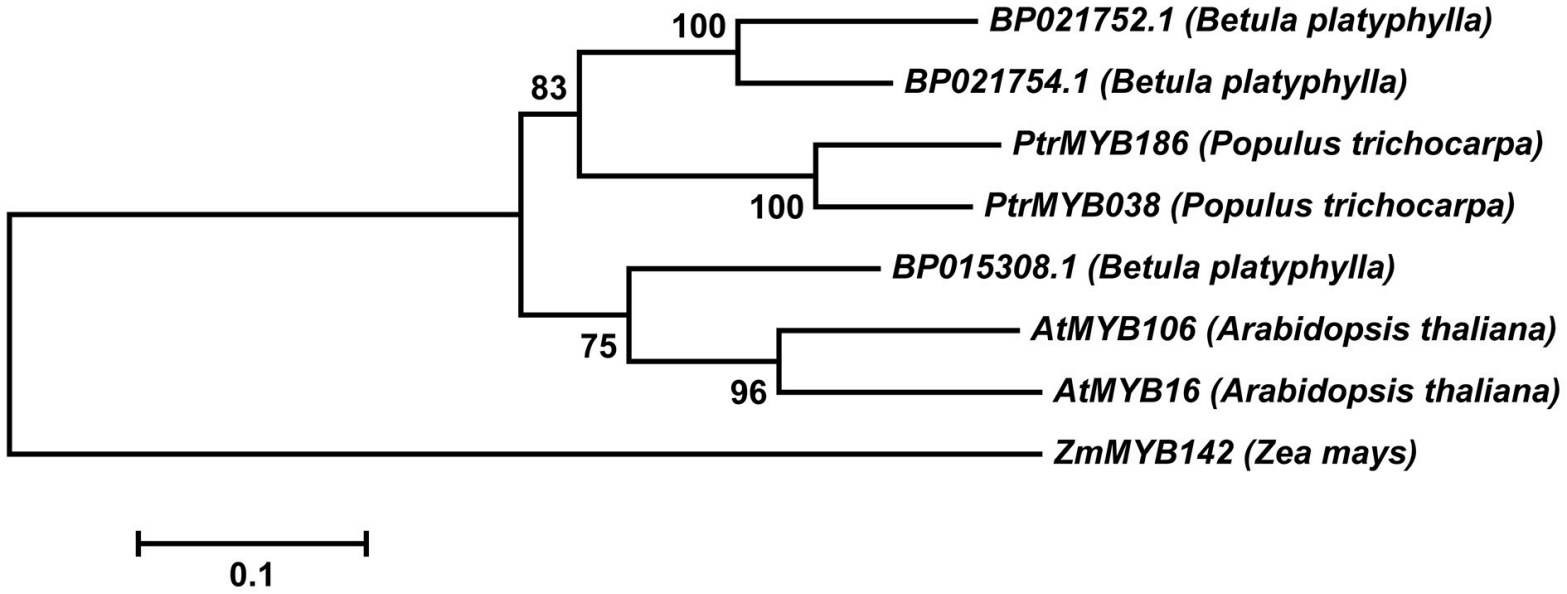
**Phylogenetic tree of R2R3-***MYB*** genes from different model plant species constructed with MEGA5 using the neighbor-joining method**. The numbers show bootstrap values for each node. Nodes with less than 50% bootstrap confidence were collapsed.

We identified *BP015308.1* (GenBank No. KR422399) from the birch genome database (http://birch.genomics.cn/page/species/index.jsp) in our study. *BP015308.1* encodes a protein of 401 amino acids (Figure S1) and belongs to R2R3-MYB transcription factors, which contain an R2R3 motif and transcription regulatory domain rich in acidic amino acid at the C-terminal ends. We selected genes related to trichome development from different model plants for multiple sequence comparison (Figure S2). BP015308.1 showed high identity (74%) with *AtMYB106* (GenBank No. AEE73615), and phylogenetic tree analysis showed that Bp015308.1 and *AtMYB106* to be in the same branch (Figure 1). Therefore, we named this gene as *BpMYB106*.

**Figure 2:**
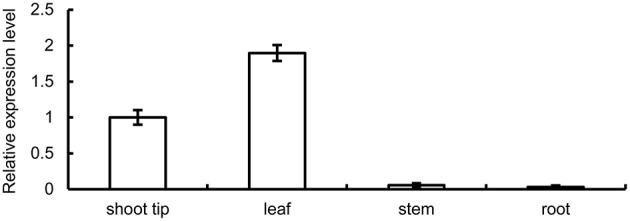
**Quantitative RT-PCR analysis of ***BpMYB106*** relative expression level in different tissues of birch**. Genes were normalized using *18S* as housekeeping genes. Error bars on each symbol indicate the mean ± SE of three replicate reactions.

We investigated the expression pattern of *BpMYB106* in different tissues using qRT-PCR. Three genes were used to choose the optimal housekeeping gene (*actin, tubulin* and *18s*). The results showed that *actin* gene expressed instability in different tissues of the birch, the specificity of *tubulin* gene is low, only *18s* gene expression is stable and has good specificity. Hence *18s* was used as RT-PCR and qRT-PCR housekeeping gene (data not shown). Highest expression of *BpMYB106* was observed in the leaves and shoot tips, and lowest in stem and root tissues (Figure 2).

### Nucleus localization of *BpMYB106*

**Figure 3:**
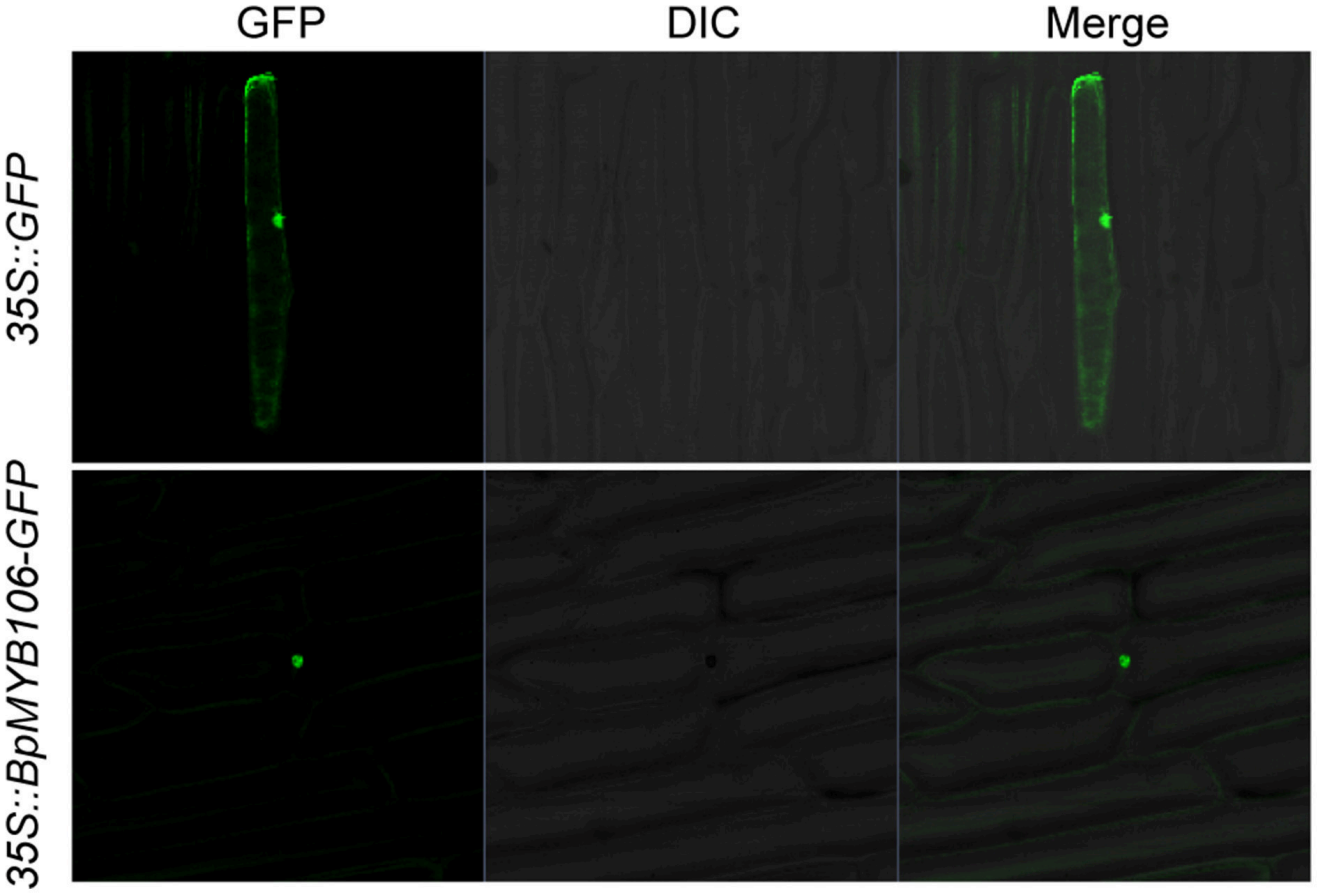
**Subcellular localization of BpMYB106**. GFP and BpMYB106-GFP fusion gene under the control of the CaMV 35S promoter were expressed transiently in onion epidermal cells. Negative controls were maintained with constructs and empty vectors. GFP, fluorescence of GFP; DIC, differential interference contrast (optical microscopic image); Merge, digital merge the images of DIC and GFP.

To observe the subcellular localization of BpMYB106 protein, we introduced the recombinant construct p35S::BpMYB106-GFP into onion (*A. cepa*) epidermal cells by particle bombardment. We observed GFP fluorescence only in the nucleus (Figure 3), whereas GFP alone showed the ubiquitous distribution in the whole cell, indicating that BpMYB106 is localized in the nucleus.

### Analysis and expression profile of *BpMYB106* promoter

Based on the cDNA sequence of *BpMYB106*, we isolated a 1588 bp promoter fragment upstream to the coding region from *B. platyphylla* by genome walking (Figure S3). Sequence analysis using PLACE showed that many putative MYB recognition sites.

Particularly, photosynthesis related or light-induced regulatory motifs corresponding to known *cis*-elements of plant genes were present in *BpMYB106* promoter, such as GATA box, I box and GT1 box (Table S1), implying that the *BpMYB106* may be associated with photosynthesis regulation.

**Figure 4:**
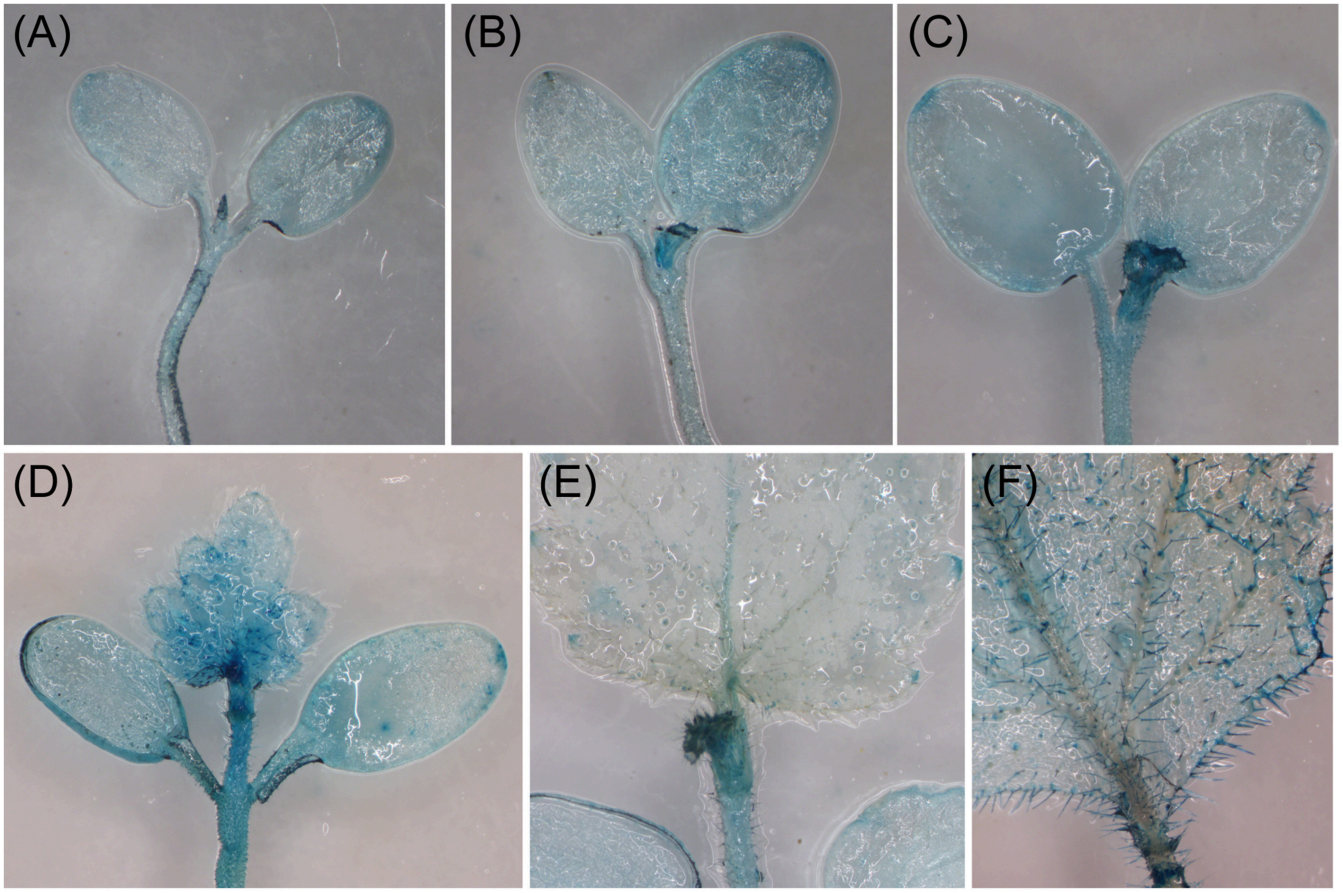
**Analysis of the expression pattern of ***BpMYB106*** in birch by the transient gene expression system. (A)**, 5-d-old seedling; **(B)**, 7-d-old seedling; **(C)**, 10-d-old seedling; **(D)**, 14-d-old seedling; **(E)**, 21-d-old seedling; **(F)**, mature leave of 50-d-old seedling.

We used *Agrobacterium*-mediated transient gene expression system to examine the expression pattern of *BpMYB106* promoter in different tissues (Zheng et al., 2012) by transiently transforming birch (at different growth stages) with the *pBpMYB106::GUS* gene. Histochemical assays showed GUS staining in shoot apical meristems, the margins of the cotyledons, and in mature leaf trichomes (Figure 4), but not in stems and roots (data not shown). The transient expression data suggests that *BpMYB106* may play a role in controlling birch shoot tip and leaf trichome development.

### Analysis of transgenic birch

**Figure 5:**
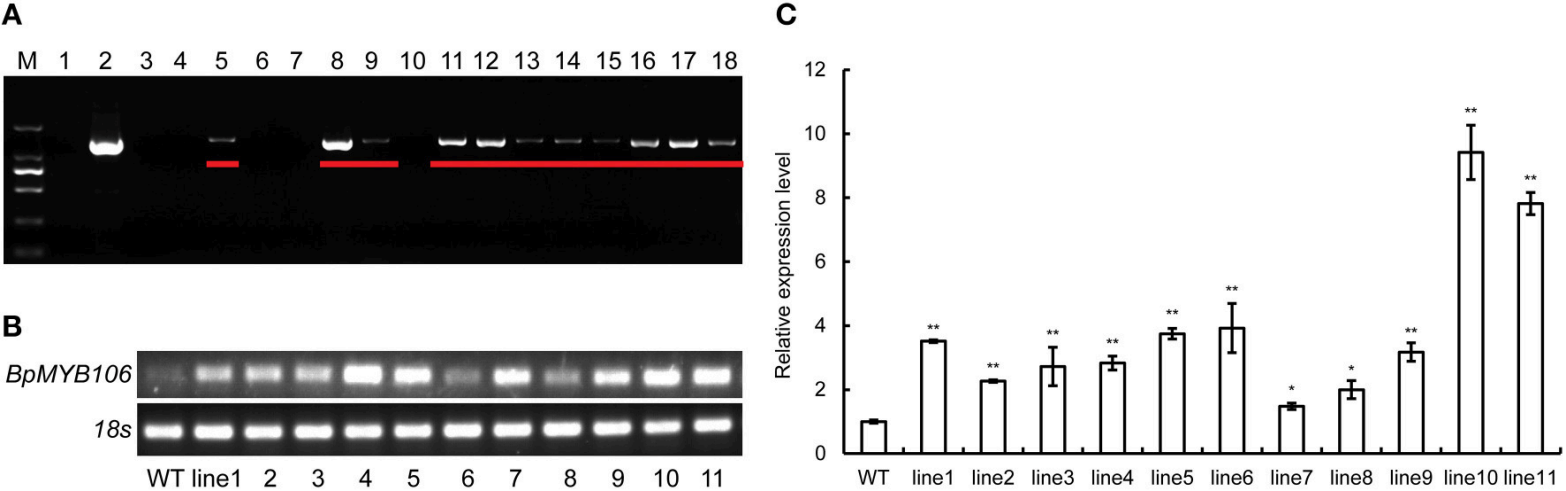
**PCR analysis of ***BMYB106*** in WT and transgenic lines of birch. (A)** PCR of *35S::BpMYB106* transgenic lines. M, DNA Marker DL2000; 1, WT plant; 2, positive control (pROKII-*BpMYB106*); 3–18, *35S::BpMYB106* transgenic lines. Underlined showed the electrophoresis result of PCR productions. **(B)** Semi-quantitative RT-PCR analysis of *BpMYB106*. WT, wild-type plant; lines 1–11; 11 PCR-positive lines. **(C)** Quantitative RT-PCR analysis of *BpMYB106*. Genes were normalized using *18S* as housekeeping genes. Error bars on each symbol indicate the mean ± SE of three replicate reactions, and statistically significant differences are indicated (^*^P < 0.05 and ^**^P < 0.01 by two-tailed *t*-test and one-way ANOVA).

We obtained 16 transgenic kanamycin resistant lines from transgenic birch constructs where the expression as driven by CaMV35S (Figure S4). PCR studies using DNA of the transformants confirmed the presence of the transgene (Figure 5A). Eleven transformed lines (lanes 5, 8, 9, and 11–18) showed the expected band size of 1206 bp indicating that the *BpMYB106* gene had been introduced into the *B. platyphylla* genome (Figure 5A). RT-PCR analysis further confirmed the presence of *BpMYB106* gene in transgenic plants (Figure 5B). qRT-PCR showed that the transcript levels of *BpMYB106* was higher in the transgenic plant lines than in WT (Figure 5C).

### Overexpression of *BpMYB106* increased foliar trichome density in birch

**Figure 6:**
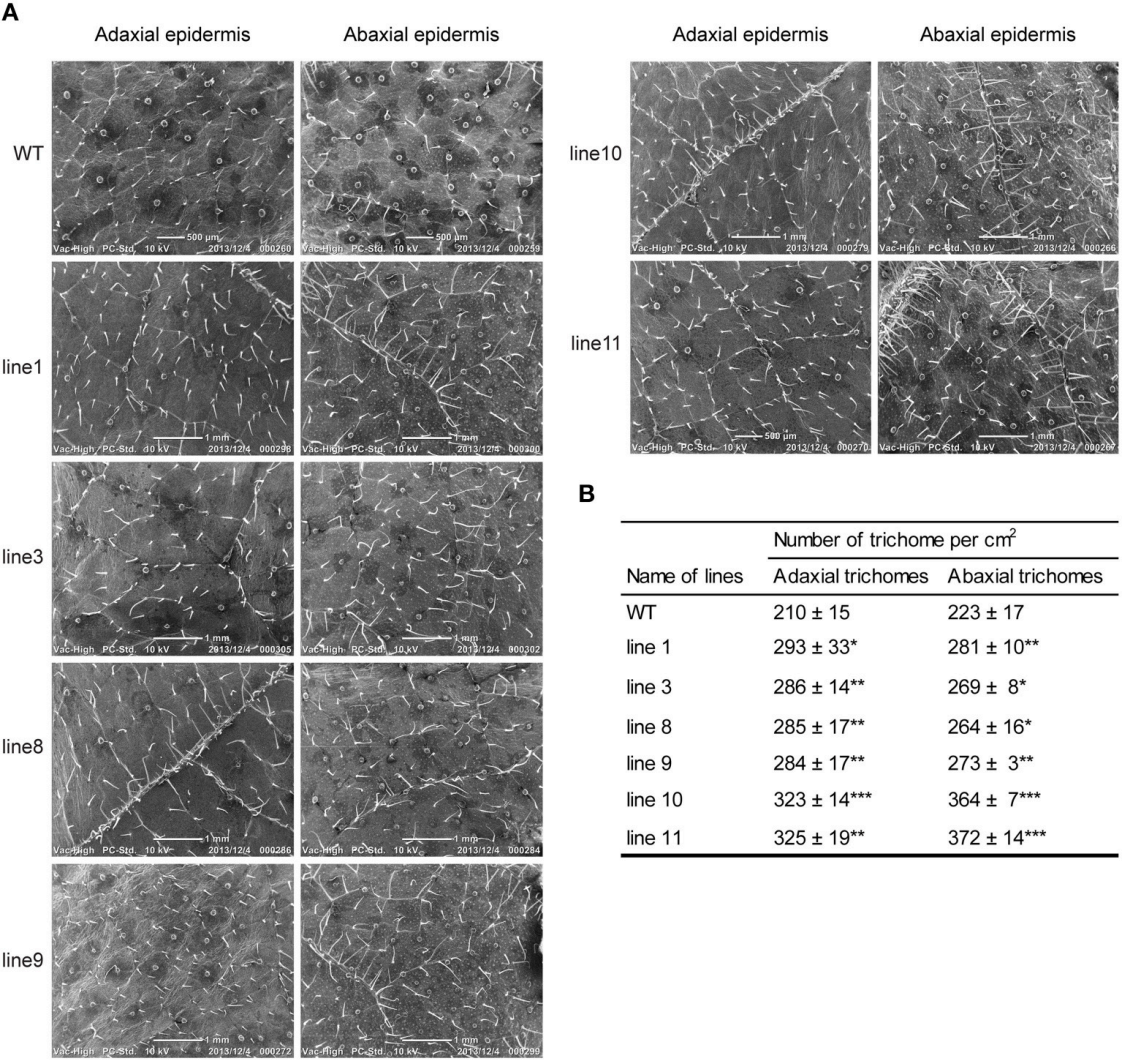
**Foliar trichome observation in WT and transgenic lines (***35S::BpMYB106***) of ***B. platyphylla*** by SEM (A) adaxial and abaxial epidermis**. **(B)** Quantity statistics of trichome between WT and transgenic lines. Errors indicate the mean ± SE of three replicate reactions, and statistically significant differences are indicated (^*^P < 0.05, ^**^P < 0.01, and ^***^P < 0.001 by two-tailed *t*-test and one-way ANOVA). WT: wild-type plant; lines 1, 3, 8, 9, 10, and 11: transgenic lines (*35S::BpMYB106*).

To examine whether the phenotypes of transgenic lines differ from their WT, we selected line 1, 3, 8, 9, 10, 11, and WT seedlings to grow both on 1/2 MS medium and in soil in the greenhouse. Scanning electron microscopy (SEM) analysis revealed that all transgenic lines had denser trichomes on both adaxial and abaxial surfaces as compared with WT plants (Figure 6).

### Overexpression of *BpMYB106* increased growth in birch

**Figure 7:**
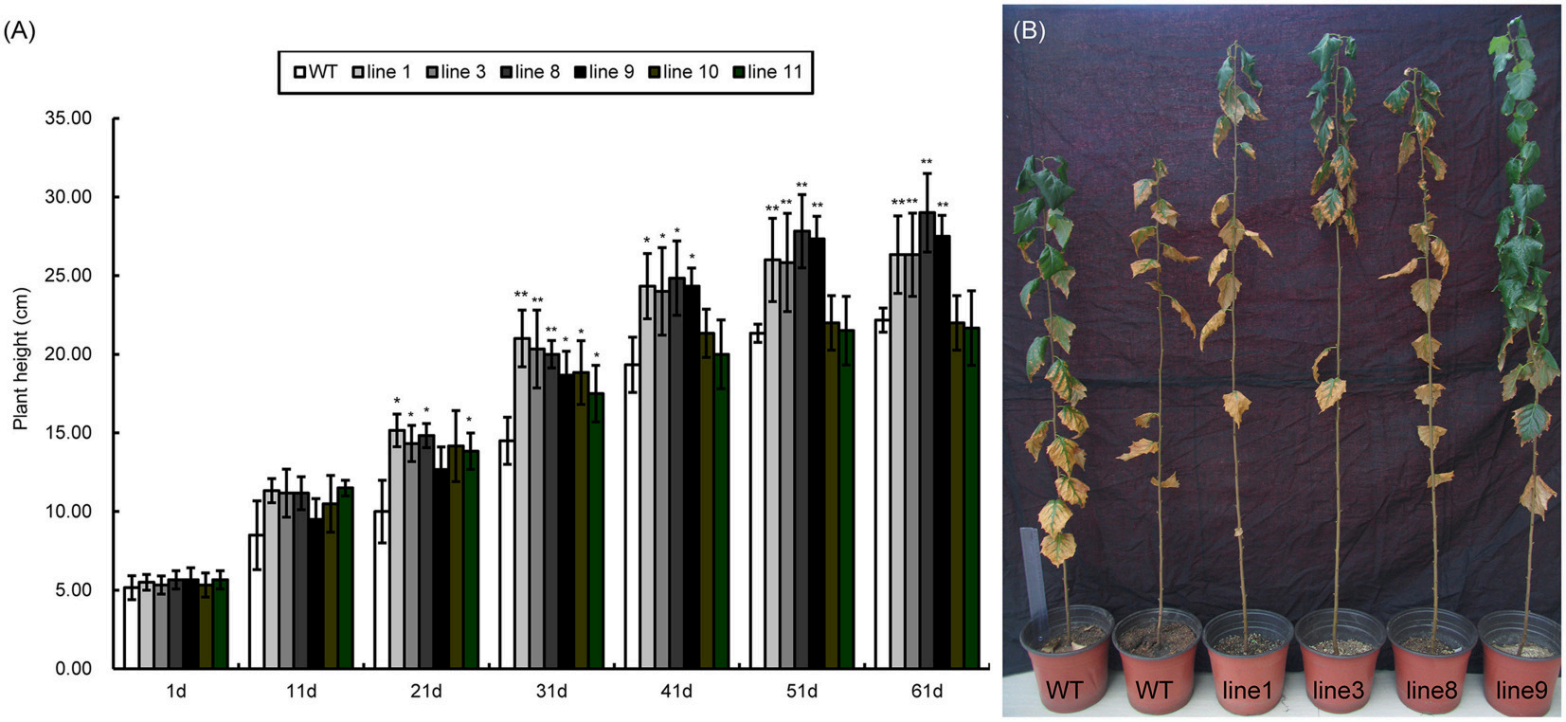
**Seedling heights of WT and transgenic lines (***35S::BpMYB106***). (A)** Seedling heights of *35S::BpMYB106* transgenic lines compared with WT plant from day 1 to 61 under glasshouse conditions. Data accompanied by the same letter indicates no significant difference, and different letters indicate a significant difference (*n* = 15, ^*^P < 0.05, ^**^P < 0.01 by two-tailed *t*-test and one-way ANOVA). Error bars on each symbol indicate the mean ± SE of three replicate reactions. **(B)** *35S::BpMYB106* transgenic lines to the right of the image shown after 8 months of growth, as compared with two WT plants on the left. WT, wild-type plant; lines 1, 3, 8, 9, 10, and 11; transgenic lines (*35S::BpMYB106*).

WT and transgenic plants showed significant differences in plant height. Both lines showed similar growth patterns in the first 1–11 days; however, the transgenic lines grew faster than the wild type after 11 days (Figure 7A). Line 1, 3, 8, and 9 did not significantly differ from each other in plant height. Interestingly, line 10 and 11 grew the same as the WT plants. Therefore, we selected four transgenic lines line 1, line 3, line 8, and line 9 (Figure 7B) that were taller than WT for studying photosynthetic efficiency.

### Overexpression of *BpMYB106* increases photosynthetic efficiency in birch

As the increase in growth increases the demand on the photosynthetic capacity of the tree (Evans, 2013). The transgenic lines line 1, line 3, line 8, and line 9 showed significantly higher rates of photosynthesis (Table 1). Transpiration rate (*E*) and stomatal conductance (*Gs*) of line 1 and line 3 was significantly higher than WT, but that of line 8 and line 9 did not significantly differ from WT. The internal CO_2_ concentration (*Ci*) of transgenic lines was also significantly lower than WT. However, transgenic lines showed higher water use efficiency (*A/E*) than WT

**Table 1:** **Analysis of the photosynthetic indexes in the wild-type and ***BpMYB106*** overexpressing transgenic birch plants grown under normal conditions**.

**Parameter**	**Wild-type**	**Line 1**	**Line 3**	**Line 8**	**Line 9**
Photosynthesis rate *A* (μmol m^−2^ s^−1^)	8.34±0.26	10.80±0.24^***^	10.96±0.45^***^	10.20±0.22^***^	10.09±0.21^***^
Stomatal conductance *Gs* (μmol m^−2^ s^−1^)	0.063±0.001	0.068±0.001^***^	0.066±0.001^***^	0.065±0.004	0.062±0.00^***^
Internal CO_2_ concentration *Ci* (μmol mol^−1^)	138.33±3.54	118.11±7.42^***^	101.63±8.97^***^	119.34±11.13^***^	109.33±4.69^***^
Transpiration rate *E* (mmol m^−2^ s^−1^)	1.87±0.003	1.99±0.03^***^	1.92±0.02^***^	1.78±0.08^***^	1.76±0.01^***^
Leaf water use efficiency (*A/E*)	4.46±0.14	5.42±0.17^***^	5.708±0.19^***^	5.75±0.21^***^	5.73±0.10^***^

WT, wild-type plant; lines 1, 3, 8, and 9: transgenic plants (35S::BpMYB106). Values represent the average of four homozygous progeny plants of each independent line. (^*^P < 0.05, ^**^P < 0.01, and

*** *P < 0.001 by two-tailed t-test and one-way ANOVA)*.

Considering the gross changes in the photosynthetic parameters of transgenic lines, we expected significant changes in their stomatal density. However, light microscopic studies did not indicate significant differences in the numbers of stomata in transgenic and WT plants (Figure S5A). Moreover, the stomatal density of transgenic lines also remained unchanged as compared to WT (*P* > 0.05, Figure S5B).

### Overexpression of *BpMYB106* increased expression of photosynthesis genes in transgenic birch

The gene expression profile from leaves of 45-day-old WT and transgenic birch (No. SRP058150) was analyzed to investigate the possible molecular mechanism of phenotype changes such as trichome density, plant growth, and photosynthetic efficiency (Table S2).

Genes with FDR ≤ 0.001 were identified as differentially expressed genes (DEGs). There were 992 DEGs between WT and line 1, including 508 upregulated, and 484 downregulated genes (Table S3). We mapped each for DEG to the Gene Ontology (GO database) for GO analysis. In total, 3192 DEGs matched genes in Blast2GO were separated into gene ontology classes according to their biological process, cellular components, and molecular function (Figure S6).

To further investigate biological behavior, we used KEGG pathway analysis to identify the biological pathways of DEGs. A total of 627 DEGs mapped into 100 pathways, and there were 20 pathways that differed most significantly between WT and line 1 (Table S4). DEGs coding for protein that are important components of photosystem II, photosystem I, cytochrome *b6f* complex and F-type ATPase were upregulated in photosynthesis and oxidative phosphorylation pathways (Table 2). We did not identify any genes typically associated with trichome development among the DEGs. However, a suite of genes with function correlated with increased trichome density were upregulated, such as genes encoding heat-shock proteins and auxin.

**Table 2:** **Differentially Expressed Genes related to photosynthesis and oxidative phosphorylation**.

**Pathway**	**Gene ID**	**log2 (line 1/WT)**	**KEGG name**	**Definition annotation**
Photosynthesis	*BP028382.1*	1.62	petD	Cytochrome *b6f* complex subunit IV, gi|460366044|
	*BP028367.1*	1.51	psbA	Photosystem II P680 reaction center D1 protein, gi|52220790|
	*BP029736.1*	1.28	ATPF1A, atpA	F-type H+-transporting ATPase subunit α, gi|290490198|
	*BP010364.1*	1.26	PsaK	Photosystem I subunit X, gi|225443988|
	*BP026585.1*	1.07	PsbW	Photosystem II PsbW protein, gi|388502126|
Oxidative phosphorylation	*BP028361.1*	5.08	COX3	Cytochrome c oxidase subunit III, gi|310757467|
	*BP028375.1*	2.51	ndhC	NAD(P)H-quinone oxidoreductase subunit III, gi|408898239|
	*BP028374.1*	2.27	ndhK	NAD(P)H-quinone oxidoreductase subunit K, gi|443267311|
	*BP028390.1*	1.58	ndhA	NAD(P)H-quinone oxidoreductase subunit I, gi|290490088|
	*BP028342.1*	1.91	ATP5G, ATP9	F-type H+-transporting ATPase subunit c, gi|388509638|
	*BP029736.1*	1.28	ATPF1A, atpA	F-type H+-transporting ATPase subunit α, gi|290490198|

**Figure 8:**
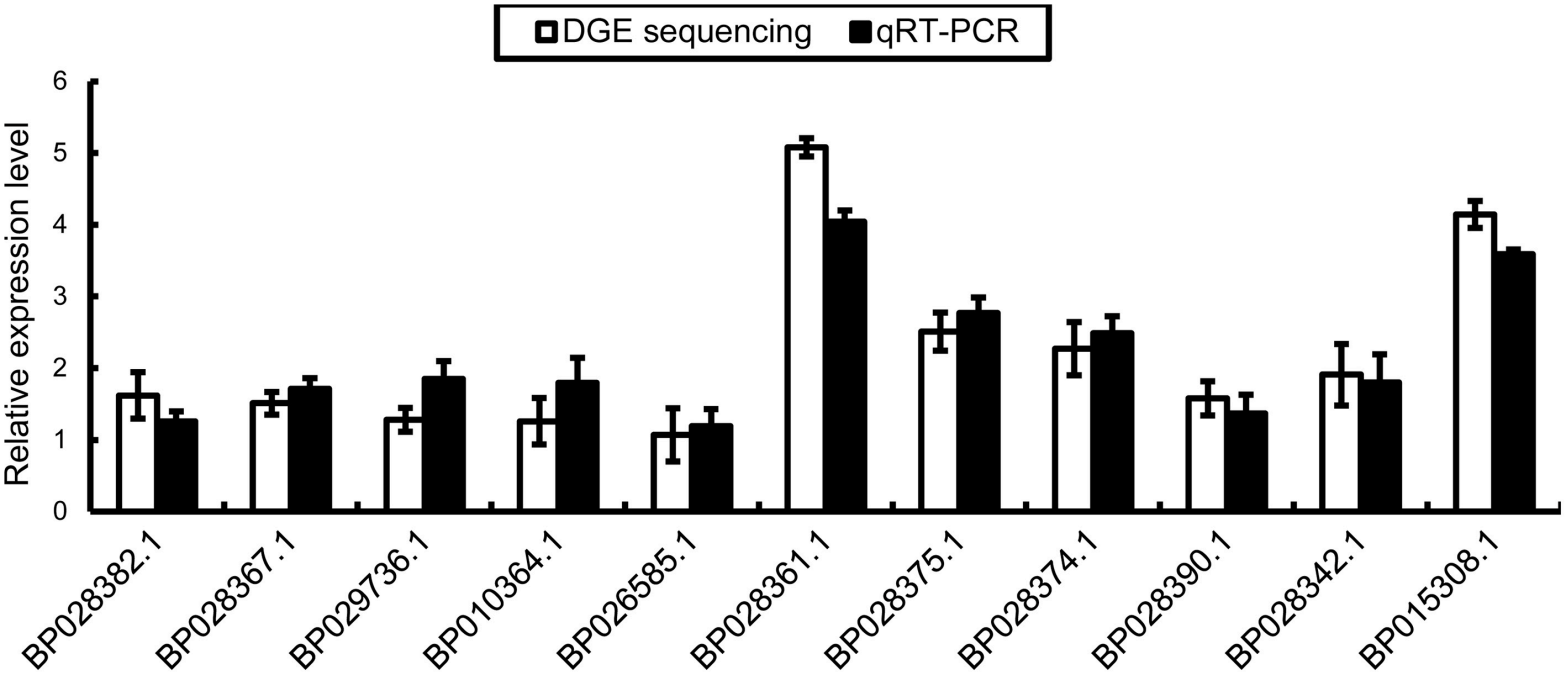
**qRT-PCR validations of DEGs in photosynthesis pathway and oxidative phosphorylation pathway**. Genes were normalized using *18S* as housekeeping genes. Error bars on each symbol indicate the mean ± SE with three replicate reactions.

We conducted qRT-PCR to validate the RNA-Seq data. We designed specific primers for *BpMYB106* (*BP018308.1*) and 10 DEGs involved in the pathway of photosynthesis and oxidative phosphorylation. The columns plot showed that the relative expression of all genes was the same as that established by RNA-Seq (Figure 8).

Promoter sequence analysis of the 10 DEGs using PLACE online database showed one common putative MYB recognition site in photosynthesis and oxidative phosphorylation genes (Table S5).

### *BpMYB106* bind several *Cis*-acting elements including MYB recognition sites and light-response elements

**Figure 9:**
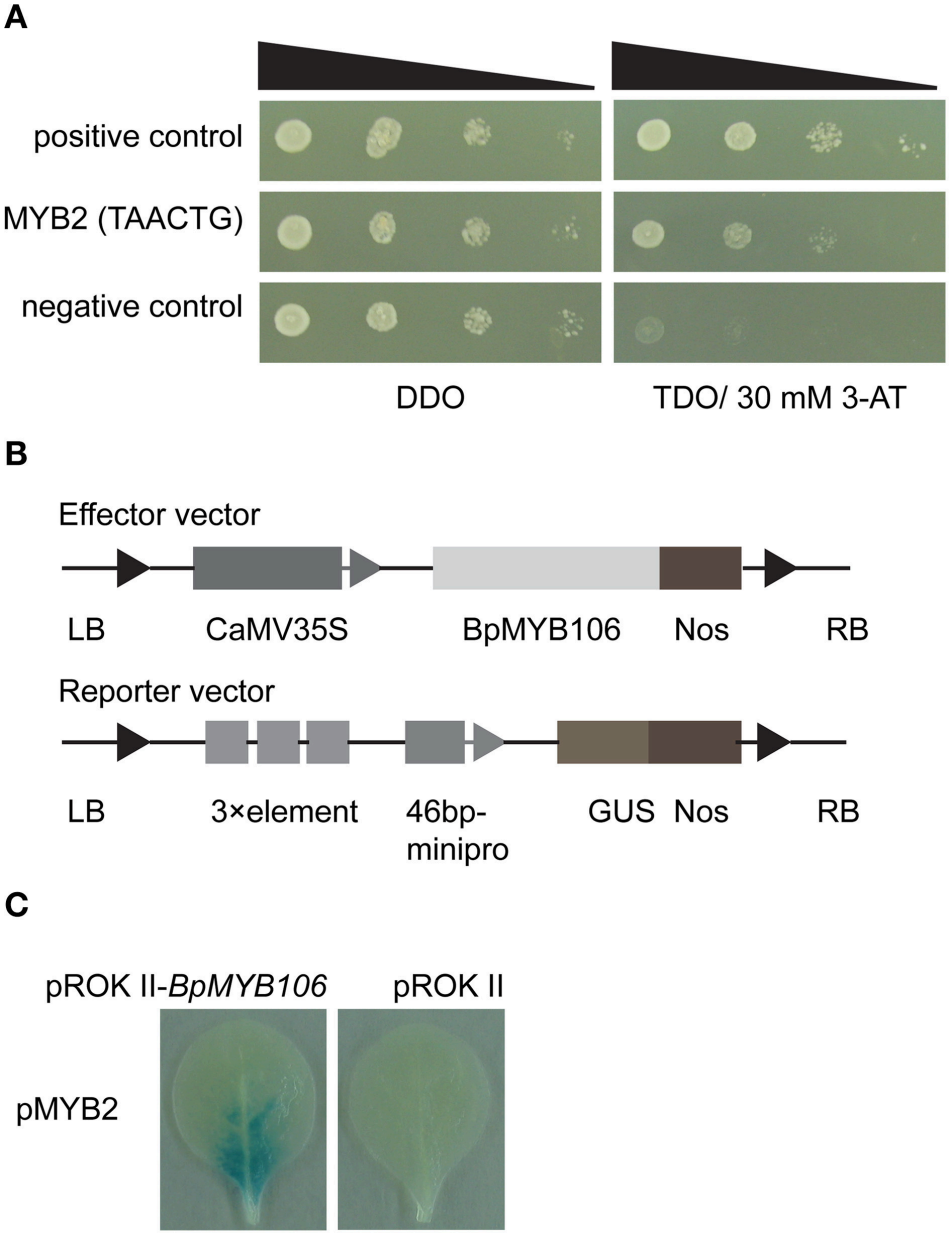
**Analyses of MYB-binding site of BpMYB106. (A)** Analysis of the binding of BpMYB106 to the MYB2 by yeast one-hybrid (Y1H) study. Yeast cells were grown on selective dropout media: SD/-Trp-Leu/-His (TDO) with 30 mM 3-amino-1, 2, 4-triazole (3-AT). Positive control: pHIS2-p53 co-transformed with pGADT7-Rec2 vector that encodes p53 fused with GAL4 AD; negative control: pHIS2-p53 co-transformed with pGADT7-Rec2-BpMYB106. **(B)** Diagram of the reporter and effector vectors used in β-glucuronidase (GUS) activity analysis. **(C)** Analysis of the interaction between MYB2 element and BpMYB106 in tobacco leaves using GUS staining, respectively. All assays were repeated three times.

We used yeast one-hybrid (Y1H) to test the binding activity of BpMYB106 to one MYB element MYB2 which was found in 10 photosynthesis and oxidative phosphorylation DEGs (Table S5). The results showed that the transformants of positive control, MYB2 element can grow on TDO medium containing 30 mM 3-AT (Figure 9A). This result suggested that the BpMYB106 transcription factor can bind MYB2 element of the 10 DEGs promoters.

The transient expression of the effector construct pROKII-*BpMYB106* (Figure 9B), and the reporter plasmids in tobacco leaves showed that BpMYB106 could bind to the MYB-related element (Figure 9C). These results suggested that BpMYB106 can bind MYB-related element MYB2 of the 10 DEG promoters correlated with photosynthesis and oxidative phosphorylation.

## Discussion

The genes encoding MYB transcription factors, that may have a role in photosynthesis, have been cloned and identified from different plants (Wang and Tobin, 1998; Churin et al., 2003; Waters et al., 2009). Most MYB genes belong to the R1/2-MYB subfamily except R2R3-MYB, which directly regulates photosynthesis. In this study, we identified a novel R2R3-MYB transcription factor BpMYB106. The function verification showed that the overexpression of BpMYB106 significantly increased trichome density, plant height, and net photosynthetic and transpiration rate in birch. We further demonstrated BpMYB106 directly regulates can photosynthesis genes to exert a growth promoting effect.

Multiple sequence comparison among several R2R3-MYB amino acid sequences showed lower sequence conservation and different functions in different species. A homologous comparison indicated that the closest *BpMYB106* sequence is orthologs with the Arabidopsis *AtMYB106* and *AtMYB16* that are related to trichome development. *AtMYB106* represses trichome outgrowth and *AtMYB16* contributes to petal form (Baumann et al., 2007; Jakoby et al., 2008). Another homologous gene *PtrMYB186* in another branch is over-expressed in poplar causing increased trichome density and enhanced poplar growth (Plett et al., 2010). Similarly, all of transgenic lines of birch (35S:*BpMYB106*) showed increased trichome density in our study.

The instantaneous net photosynthesis rate measures plant photosynthetic efficiency most efficiently and directly. Even small increases in the rate of net photosynthesis can translate into large increases in biomass, and hence yield (Parry et al., 2011). In our study, all transgenic lines of birch with improved growth rate showed significantly increased net photosynthesis rate (*A*) and leaf water use efficiency (*A/E*), associated with increased plant height. The reduced internal CO_2_ concentration (*Ci*) and increased transpiration rate (*E*) of line 1 and line 3 indicated that the overall photosynthesis level of transgenic lines was higher than WT. Stomata—an important index in photosynthesis measurement—handle gas exchange and transpiration of water (McLachlan et al., 2014). WT and transgenic lines did not show significant differences in stomatal density. However, line 1 and line 3 showed higher stomatal conductance (*Gs*), which can be attributed to a decrease in the resistance to CO_2_ leading to a higher rate of photosynthesis (Plett et al., 2010). According to the photosynthesis indices, the photosynthetic capacity of transgenic lines was higher than WT, at least in line 1 and line 3. There may be differences in measuring data among transgenic lines due to individual variations, yet most of the photosynthesis indexes indicate the superior photosynthetic capacity of transgenic lines.

Among the many factors that influence photosynthesis, photosynthetic gene expression is a key factor (Yu et al., 2014). Our RNA-Seq analysis showed that five DEGs function in the photosynthesis pathway. These DEGs are associated with photosystem II, photosystem I, cytochrome *b6f* complex and F-type ATPase. They are essential and related to the utilization of light energy (Takabe et al., 1991; Green and Hollingsworth, 1992; Zhang et al., 2014). The deletion of tobacco *psbA* gene coding core components of photosystem II (PSII) downregulates specific groups of nuclear and chloroplast genes involved in photosynthesis, energy metabolism, and chloroplast biogenesis (Leelavathi et al., 2011). In our study, RNA-Seq analysis also showed that many DEGs participate in oxidative phosphorylation (Table 2), which is crucial for plant growth (Reynolds et al., 2012). The mitochondria carry out the final steps of oxidative phosphorylation and generate the bulk of the ATP in respiration, which provides energy for biosynthesis (Jacoby et al., 2012). Moreover, its balance with photosynthesis determines the rate of plant biomass accumulation (Millar et al., 2011). The elevated photosynthetic rate in transgenic birch lines may be because of the upregulation of the photosynthesis and oxidative phosphorylation genes. *Fv/Fm* of leafs was mesured at the same time of photosynthesis indexes measuring, but the statistical analysis result showed there was no significant differences in *Fv/Fm* between wild-type and transgenic lines of birch (Table S10). In generally, the *Fv/Fm* is a important indicator to detemine the ability of plant stress response, and qRT-PCR further proved that 10 of these DEGs were up regulated in line 1. Y1H and transient assays showed that BpMYB106 directly binds to the *cis*-acting element MYB2 which was in those DEGs promoters. Thus, BpMYB106 may directly activate the expression of a range of photosynthesis and oxidative phosphorylation related genes by interacting with MYB2 element in their promoters (Ji et al., 2014).

The nuclear genome of eukaryotes contains large amounts of cytoplasmic organelle DNA (nuclear integrants of organelle DNA [*norgs*]) (Rousseau-Gueutin et al., 2011). Presence of chloroplast-related DNA sequences in the nuclear genome is generally regarded as a relic of the process by which genes have been transferred from the chloroplast to the nucleus (Cullis et al., 2009). The transfer to the nuclear genome of most of the protein-encoding functions for chloroplast located proteins facilitates the control of gene expression. This phenomenon was observed in tobacco (Ayliffe and Timmis, 1992) and rice (Akbarova et al., 2010). In this study, subcellular localization indicated BpMYB106 was a predicted nucleus transcript factor, but interestingly, RNA-Seq data showed several genes affected are plastid transcripts, of which have been identified in chloroplast genome of *Arabidopsis* (Sato et al., 1999). These DEGs (Table 2) were found in birch genome database (has not yet release publicly). Coincidentally, the homologous genes were found in *Populus trichocarpa* chromosome genome, such as Potri.013G138300 (GenBank No. A4GYN9) which is photosystem II reaction center protein, Potri.013G137900 (GenBank No. A4GYP4) which is ATPase subunit. The changes in chloroplast-related transcripts could be secondary effects of changing a nuclear-based gene that affects chloroplast processes. The phenomenon in birch may provide some reference for study of *norgs* in woody plants.

Although, transgenic lines in our study were taller than WT, RNA-Seq data did not show up regulation of genes typically associated with increased growth such as cell wall synthesis or cell cycling genes. Thus, the increased photosynthesis as a result of up regulated photosynthetic genes (Evans, 2013) may be the sole factor affecting plant growth. This approach is worth exploring further to increase growth rate of birch. Improved photosynthesis in transgenic birch may be because of increased trichome density (Plett et al., 2010). Interestingly, our study shows that “the more, the better” is not true for trichome density. We observed the highest trichome density in lines line 10 and line 11 where *BpMYB106* expression was the highest. However, these plants were not significantly taller than other transgenics or even WT. Perhaps, a certain trichome density helps in the improvement of plant growth in birch. However, we did not find genes related to trichome development in our RNA-Seq data as compared with the whole-transcriptome result of *fuzzy* mutant. The primary reason could be that the functional leaf samples used for RNA-Seq had matured trichomes.

In summary, BpMYB106 promotes trichome density, improves net photosynthesis rate, and plant growth in birch. Gene expression profile revealed that the overexpression of BpMYB106 regulates many photosynthesis related genes. Y1H assay and transient assay showed that BpMYB106 directly binds to the *cis*-acting element MYB2 in a range of photosynthesis related genes promoters. Our study enriches the research of R2R3-MYB transcription factors function and provides a basis for fundamental studies on birch molecular breeding. These results demonstrating that increasing transcript level of an R2R3-MYB transcription factor, BpMYB106, improves photosynthesis and growth rate in birch via up regulating genes of photosynthesis and oxidative phosphorylation.

## Author contributions

Conceived and designed the experiments: CZ, CL. Performed the experiments: CZ. Analyzed the data: CZ, CL. Wrote the paper: CZ, CL.

### Conflict of interest statement

The authors declare that the research was conducted in the absence of any commercial or financial relationships that could be construed as a potential conflict of interest.

## Supplementary material

Figure S1 **Nucleotide and amino acid sequence of *BpMYB106***. Gray sections indicate conserved domain of R2R3-MYB DNA binding sites.

Figure S2 **Multiple-sequence alignment of R2R3-MYB proteins from different model plant species**. A conserved motif of the R2R3 domain is boxed with black lines.

Figure S3 **Nucleotide sequence of BpMYB106 promoter**. Lined section indicates BpMYB106 gene sequence contained initiation site “ATG.”

Figure S4 **Regeneration and identification of *BpMYB106* transgenic lines. (A,B)** Leaf and stem explants. Red arrows indicated the transgenic callus sites. **(C)** Transgenic cluster of shoots has formed from leaf callus. **(D)** Transgenic callus formed on one of the cut sites in a stem segment. **(E)** A transgenic cluster of shoots cultured in differential medium for 15 days. **(F)** Transgenic shoots transferred to rooting medium.

Figure S5 **Stomatal density investigation in WT and transgenic lines (*35S::BpMYB106*). (A)** Stomatal density of leaf abaxial surface in WT and transgenic lines of line 1, line 3, line 8, and line 9. The black bar indicated the length of 100 μm. **(B)** Quantity statistics of stoma between WT and transgenic lines. Error bars on each symbol indicate the mean ± SE of three replicate reactions.

Figure S6 **GO functional classification of differentially expressed genes (DEGs)**.

Table S1 ***cis*-acting elements analysis of *BpMYB106* promoter by PLACE**.

Table S2 **Summary statistics of sequencing in WT and transgenic line (*35S::BpMYB106*) of *B. platyphylla***.

Table S3 **Differentially expressed genes between WT and transgenic line (*35S::BpMYB106*)**.

Table S4 **Twenty pathways that differed most significantly between WT and transgenic line (*35S::BpMYB106*)**.

Table S5 **Analysis of MYB binding site of 10 DEGs promoters using PLACE**.

Table S6 **List of primers used for the cloning of cDNAs and plasmid constructions**.

Table S7 **List of primers used for the quantitative RT-PCR**.

Table S8 **List of primers used for Y1H**.

Table S9 **List of primers used for the transient assay**.

Table S10 **Maximum quantum yield of PSII (*Fv/Fm*) of WT and transgenic lines**.
