# Supplementary figures and images for: A Novel R2R3-MYB Transcription Factor BpMYB106 of Birch (Betula platyphylla) Confers Increased Photosynthesis and Growth Rate through Up-regulating Photosynthetic Gene Expression
Source: Front Plant Sci. 2016 Mar 22;7:315. doi: 10.3389/fpls.2016.00315 (PMC4801893; doi:10.3389/fpls.2016.00315)

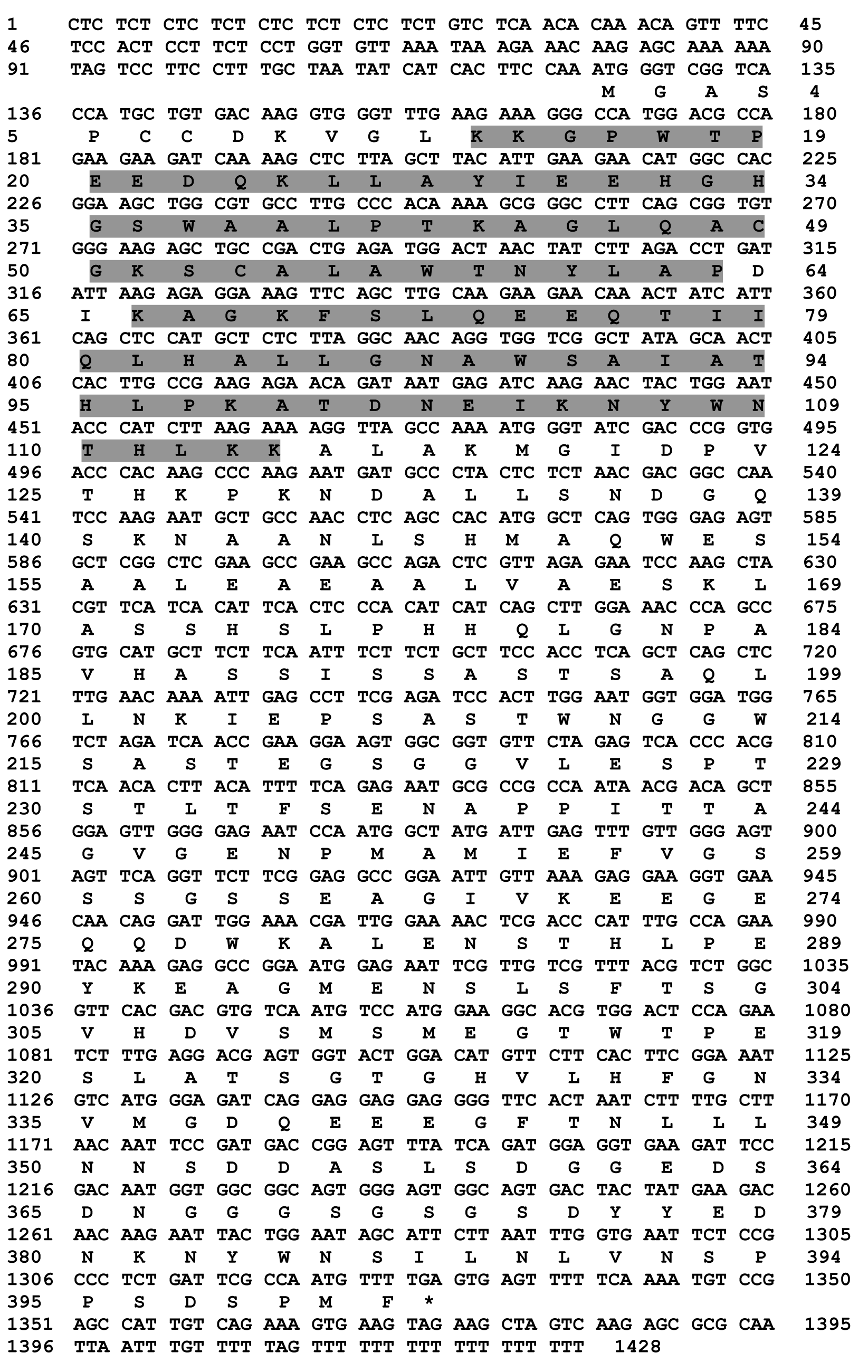

Supplement: Figure S1 — Nucleotide and amino acid sequence of BpMYB106. Gray sections indicate conserved domain of R2R3-MYB DNA binding sites. [file Image1.TIF]

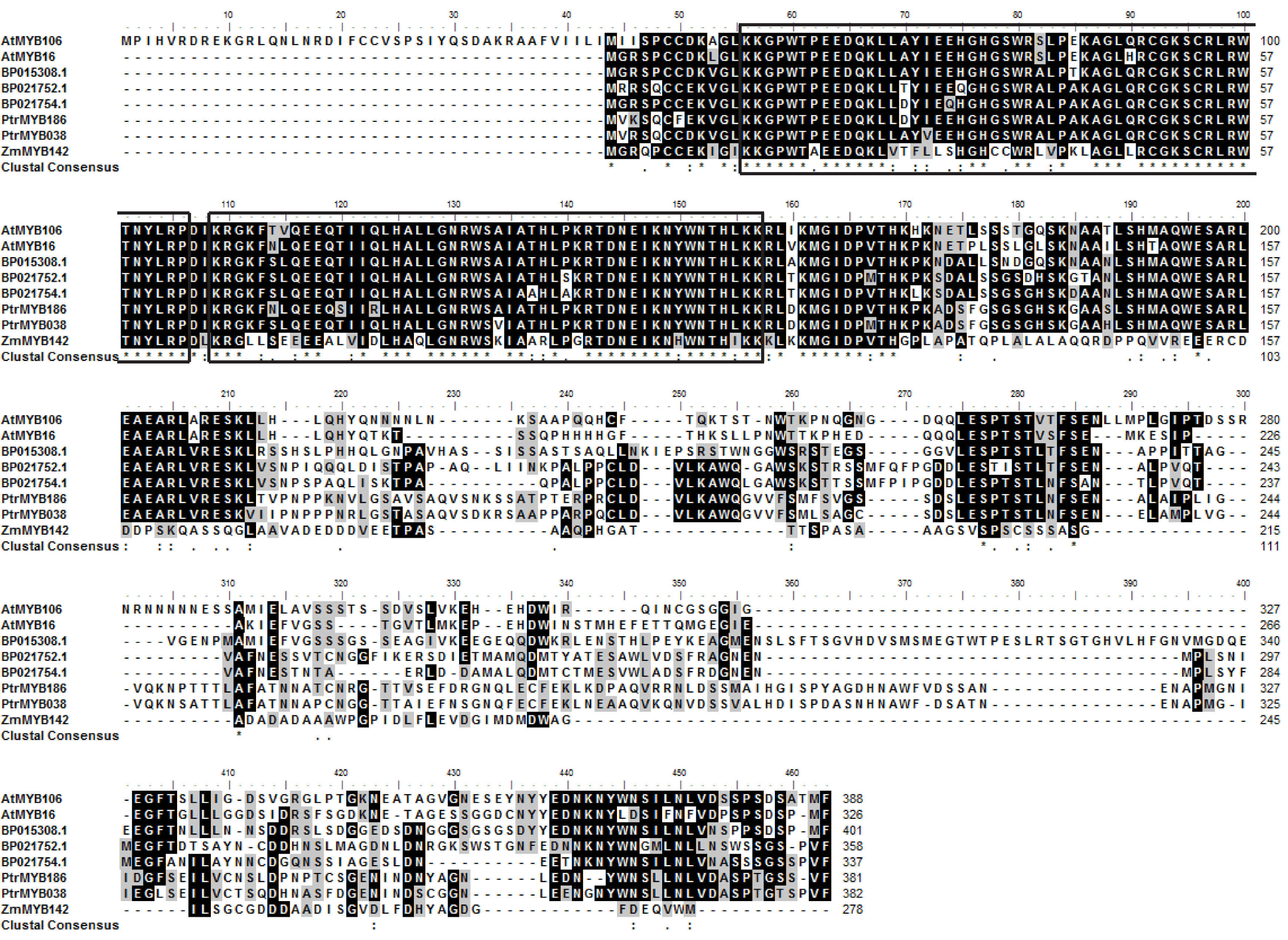

Supplement: Figure S2 — Multiple-sequence alignment of R2R3-MYB proteins from different model plant species. A conserved motif of the R2R3 domain is boxed with black lines. [file Image2.TIF]

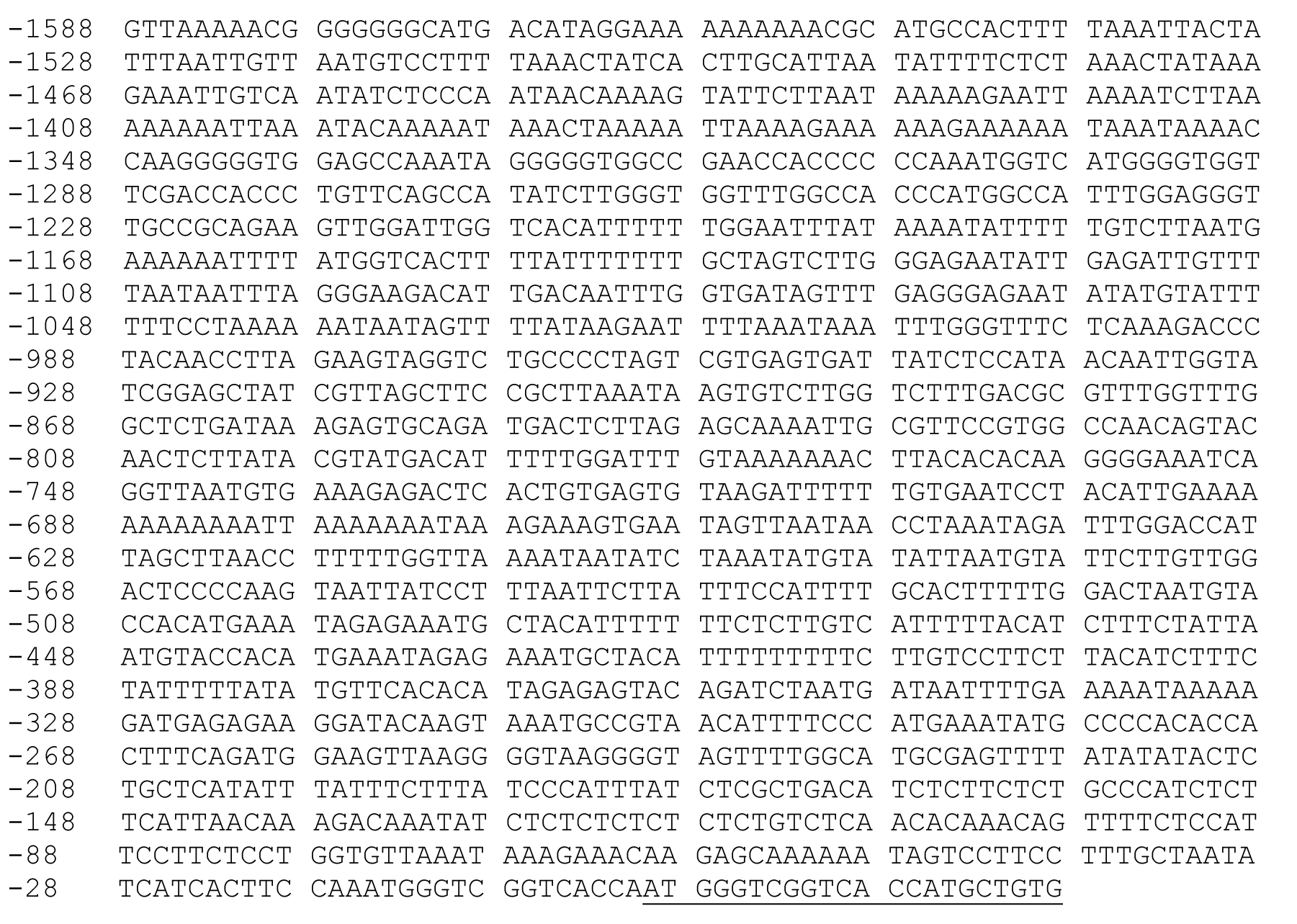

Supplement: Figure S3 — Nucleotide sequence of BpMYB106 promoter. Lined section indicates BpMYB106 gene sequence contained initiation site “ATG.” [file Image3.TIF]

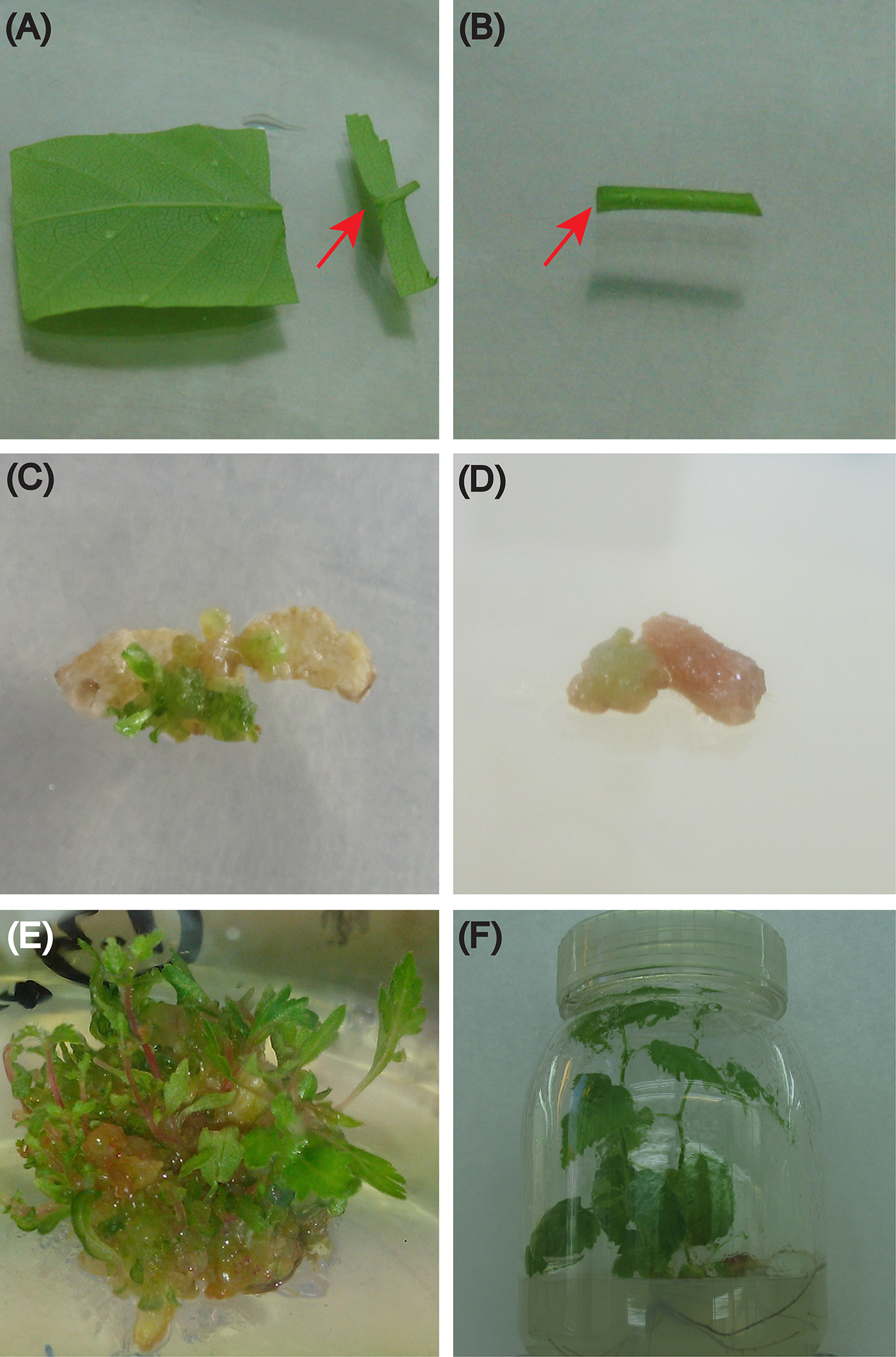

Supplement: Figure S4 — Regeneration and identification of BpMYB106 transgenic lines. (A,B) Leaf and stem explants. Red arrows indicated the transgenic callus sites. (C) Transgenic cluster of shoots has formed from leaf callus. (D) Transgenic callus formed on one of the cut sites in a stem segment. (E) A transgenic cluster of shoots cultured in differential medium for 15 days. (F) Transgenic shoots transferred to rooting medium. [file Image4.TIF]

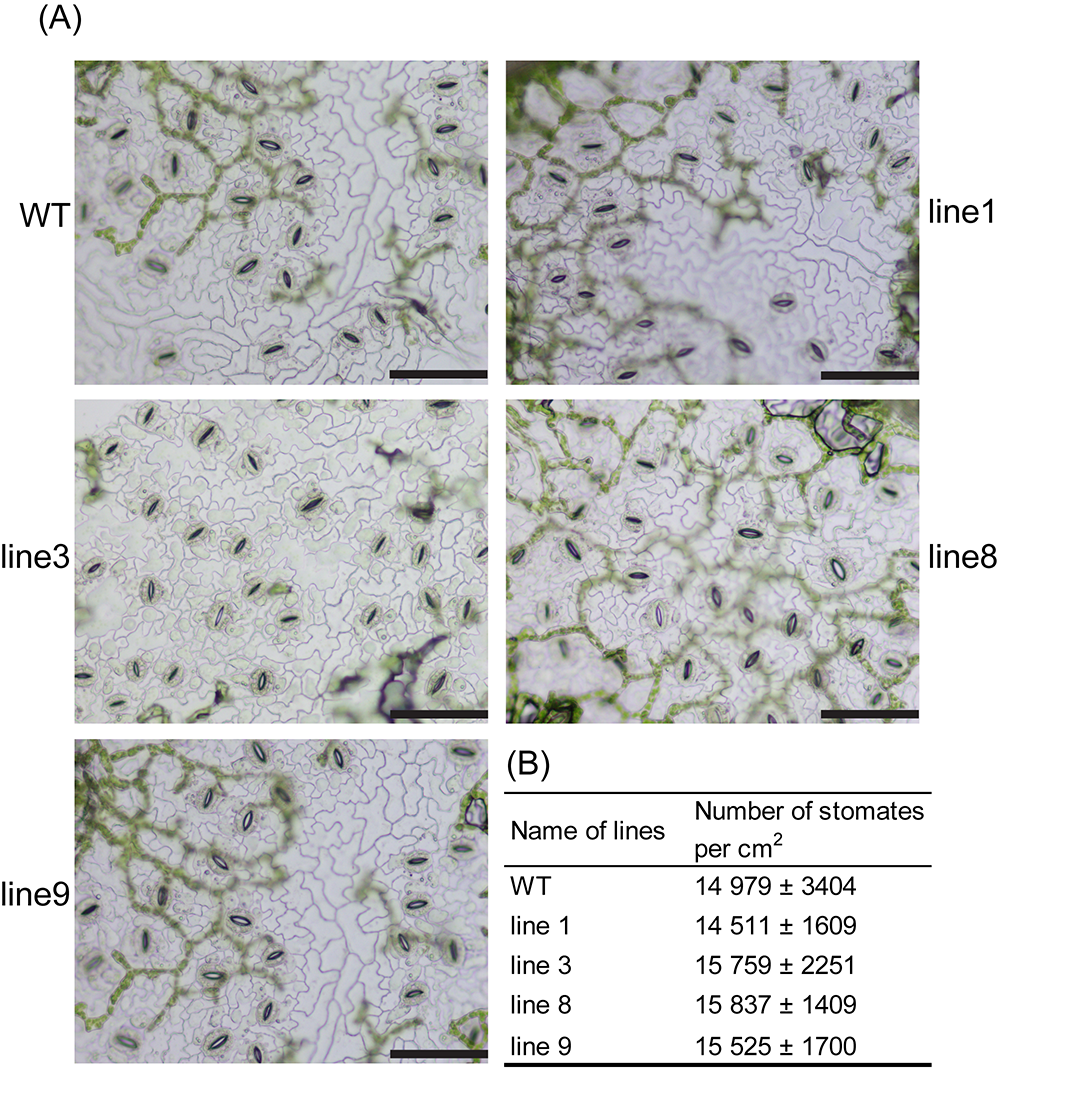

Supplement: Figure S5 — Stomatal density investigation in WT and transgenic lines (35S::BpMYB106). (A) Stomatal density of leaf abaxial surface in WT and transgenic lines of line 1, line 3, line 8, and line 9. The black bar indicated the length of 100 μm. (B) Quantity statistics of stoma between WT and transgenic lines. Error bars on each symbol indicate the mean ± SE of three replicate reactions. [file Image5.TIF]

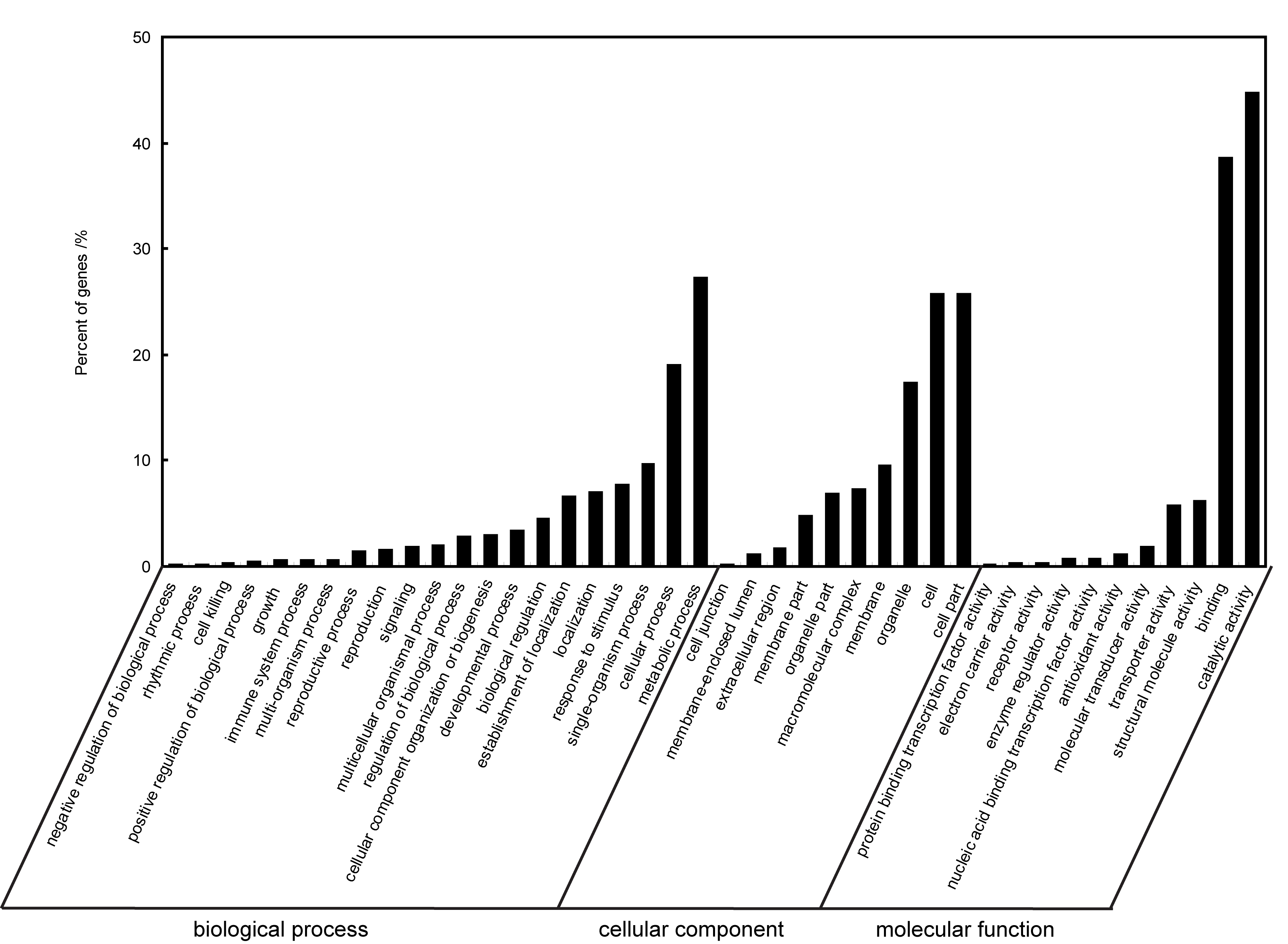

Supplement: Figure S6 — GO functional classification of differentially expressed genes (DEGs). [file Image6.TIF]
