# Supplementary material for: A Novel R2R3-MYB Transcription Factor BpMYB106 of Birch (Betula platyphylla) Confers Increased Photosynthesis and Growth Rate through Up-regulating Photosynthetic Gene Expression
Source: Front Plant Sci. 2016 Mar 22;7:315. doi: 10.3389/fpls.2016.00315 (PMC4801893; doi:10.3389/fpls.2016.00315)
Supplement: Table S1 — cis-acting elements analysis of BpMYB106 promoter by PLACE. [file Table1.DOC]

Table S1 cis-acting elements analysis of BpMYB106 promotor by PLACE (http://www.dna.affrc.go.jp/PLACE/).

| Name | Sequence | Position | Description |
| --- | --- | --- | --- |
| CATA | YACT | -9 (+); -198 (+); -255 (+); -512 (+); -714 (+); -1138 (+); -1485 (+); -296 (-); -348 (-); -545 (-); -649 (-); -707 (-); -798 (-); -841 (-); -883 (-); -939 (-); -961 (-); -1425 (-) | A key component of Mem1 (mesophyll expression module 1) found in the *cis*-regulatory element in the distal region of the phosphoenolpyruvate carboxylase (ppcA1) of the C4 dicot *F. trinervia* |
| GATA BOX | GATA | -302 (+); -334 (+); -848 (+); -1061 (+); -15 (-); -116 (-); -166 (-); -175 (-); -539 (-); -587 (-); -906 (-); -915 (-); -933 (-); -1253 (-); -1442 (-); -1488 (-) | Three GATA box repeats were found in the promoter of Petunia chlorophyll a/b binding protein, Cab22 gene; Required for high level, light regulated, and tissue specific expression. |
| I box | GATAA | -334 (+); -848 (+); -167 (-); -176 (-); -540 (-); -934 (-) | Conserved sequence upstream of light-regulated genes of both monocots and dicots |
| SORLIP | GCCAC | -1237 (+); -1309 (-); -807 (-) | Sequences Over-Represented in Light-Induced Promoters (SORLIPs) is most over-represented, and most statistically singnifican. |
| GT-1 | GRWAAW | -325 (+); -334 (+); -678 (+); -741 (+); -848 (+); -1154 (+); -1350 (+); -1357 (+); -168 (-); -177 (-); -280 (-); -281 (-); -399 (-); -466 (-); -524 (-); -541 (-); -935 (-); -1034 (-); -1035 (-); -1472 (-) | Consensus GT-1 binding site in many light-regulated genes, GT-1 can stabilize the TFIIA-TBP-DNA (TATA box) complex. |
| GT-1 CORE | GGTTAA | -598 (+); -733 (+); -609 (-) | Critical for GT-1 binding to box II of rbcS |
| SV40 core enhancer | GTGGWWHG | -1245 (+); -1278 (+); -1304 (-) | Similar sequences found in rbcS genes |
| rbcS | AATCCAA | -770 (+); -1201 (+) | rbcS general consensus sequence |
| MYB1 | WAACCA | -1337 (+); -599 (-); -860 (-); -1244 (-) | Regulating many aspects of plant development, including epidermis or trichome development, flavonoid biosynthesis, or ABA –response gene expression. |
| MYBCORE | CNGTTR | -802 (-) |
| MYBGAHV | TAACAAA | -128 (+); -1432 (+) |
| MYBPLANT | MACCWAMC | -863 (-) |
| MYBST1 | GGATA | -303 (+); -175 (-); -539 (-) |
