# Supplementary material for: A Novel R2R3-MYB Transcription Factor BpMYB106 of Birch (Betula platyphylla) Confers Increased Photosynthesis and Growth Rate through Up-regulating Photosynthetic Gene Expression
Source: Front Plant Sci. 2016 Mar 22;7:315. doi: 10.3389/fpls.2016.00315 (PMC4801893; doi:10.3389/fpls.2016.00315)
Supplement: Table S2 — Summary statistics of sequencing in WT and transgenic line (35S::BpMYB106) of B. platyphylla. [file Table2.DOC]

Table S2 Summary statistics of sequencing in wild-type and transgenic line (*35S::BpMYB106*) of *B. platyphylla*.

|  |  | WT | | OE | |
| --- | --- | --- | --- | --- | --- |
|  |  | reads number | percentage | reads number | percentage |
| Map to Gene | Total Reads | 6065504 | 100.00% | 6157633 | 100.00% |
|  | Total BasePairs | 297209696 | 100.00% | 301724017 | 100.00% |
|  | Total Mapped Reads | 3695439 | 60.93% | 3620282 | 58.79% |
|  | perfect match | 2727906 | 44.97% | 2698037 | 43.82% |
|  | <=2bp mismatch | 967533 | 15.95% | 922245 | 14.98% |
|  | unique match | 3041610 | 50.15% | 2968266 | 48.20% |
|  | multi-position match | 653829 | 10.78% | 652016 | 10.59% |
|  | Total Unmapped Reads | 2370065 | 39.07% | 2537351 | 41.21% |
|  | Number of unigenes | 3022644 | - | 2950714 | - |
| Map to Genome | Total Reads | 6065504 | 100.00% | 6157633 | 100.00% |
|  | Total BasePairs | 297209696 | 100.00% | 301724017 | 100.00% |
|  | Total Mapped Reads | 4821210 | 79.49% | 4935366 | 80.15% |
|  | perfect match | 3388264 | 55.86% | 3501538 | 56.86% |
|  | <=3bp mismatch | 1432946 | 23.62% | 1433828 | 23.29% |
|  | unique match | 3712179 | 61.20% | 3721364 | 60.43% |
|  | multi-position match | 1109031 | 18.28% | 1214002 | 19.72% |
|  | Total Unmapped Reads | 1244294 | 20.51% | 1222267 | 19.85% |
|  | Number of genes | 19726 | - | 20184 | - |
