# Supplementary material for: A Novel R2R3-MYB Transcription Factor BpMYB106 of Birch (Betula platyphylla) Confers Increased Photosynthesis and Growth Rate through Up-regulating Photosynthetic Gene Expression
Source: Front Plant Sci. 2016 Mar 22;7:315. doi: 10.3389/fpls.2016.00315 (PMC4801893; doi:10.3389/fpls.2016.00315)
Supplement: Table S4 — Twenty pathways that differed most significantly between WT and transgenic line (35S::BpMYB106). [file Table4.DOC]

| Pathway | Sample1 (627) | Sample2 (16823) | Pvalue | Qvalue | Pathway ID | Genes | KOs |
| --- | --- | --- | --- | --- | --- | --- | --- |
| Ribosome | 50 | 338 | 5.20E-17 | 5.20E-15 | ko03010 | BP028384.1;BP028385.1;BP034467.1;BP029735.1;BP036062.1;BP028366.1;BP031022.1;BP006690.1;BP031027.1;BP025488.1;BP029246.1;BP025506.1;BP005046.1;BP028373.1;BP031465.2;BP020116.2;BP008902.1;BP020553.1;BP010566.1;BP007010.1;BP012027.1;BP034937.1;BP034241.1;BP012457.1;BP030290.1;BP025094.1;BP020881.1;BP020770.1;BP009687.1;BP021166.1;BP016278.1;BP021722.1;BP005190.1;BP014501.1;BP001666.1;BP012633.1;BP026407.1;BP008966.1;BP031441.1;BP019820.1;BP021089.1;BP003968.1;BP027536.1;BP010078.1;BP008830.1;BP007257.1;BP036775.2;BP019263.1;BP003134.1;BP033603.1 | K02874+K02878+K02934+K02967+K02920+K02886+K02971+K02866+K02983+K02919+K02896+K02986+K02975+K02875+K02991+K02915+K02918+K02993+K02995+K02912+K02939+K02957+K02980+K02964+K02974+K02923+K02973+K02917+K02951+K02989+K02894+K02921+K02889+K02880+K02910+K02949+K02936 |
| Plant-pathogen interaction | 118 | 1763 | 1.34E-10 | 6.70E-09 | ko04626 | BP030562.1;BP028202.1;BP028215.1;BP028212.1;BP008343.1;BP034354.1;BP029415.1;BP009006.1;BP021681.1;BP002719.1;BP031854.1;BP008289.1;BP010788.1;BP023703.8;BP010645.1;BP005223.1;BP015494.1;BP034495.3;BP030133.1;BP029457.1;BP017916.1;BP037042.1;BP030124.1;BP005978.1;BP016631.1;BP021442.3;BP013727.1;BP030126.1;BP001783.1;BP014265.2;BP008286.1;BP011193.1;BP009331.1;BP001098.1;BP029463.1;BP008659.1;BP019970.1;BP014705.4;BP031270.1;BP021571.1;BP006827.1;BP029461.1;BP005800.1;BP030131.1;BP014056.1;BP031855.2;BP015109.4;BP001093.1;BP005980.1;BP023343.1;BP031621.1;BP029356.1;BP006372.1;BP036878.1;BP036884.1;BP015491.1;BP033319.1;BP008288.1;BP037189.1;BP023316.1;BP021540.1;BP023347.1;BP029355.1;BP017590.1;BP004602.1;BP013726.1;BP020437.1;BP007736.1;BP017917.1;BP022716.1;BP011551.1;BP015421.1;BP004604.1;BP029917.1;BP033244.1;BP029574.1;BP010212.1;BP020436.1;BP027938.1;BP001454.1;BP035186.1;BP035218.1;BP023349.1;BP014580.1;BP028486.2;BP009321.1;BP022333.2;BP006968.1;BP008986.1;BP030632.1;BP002355.1;BP000028.1;BP005106.1;BP009335.2;BP013732.2;BP016559.1;BP023699.4;BP027935.2;BP019969.1;BP009319.1;BP034358.3;BP008146.1;BP037197.1;BP023697.1;BP034951.1;BP004880.2;BP007099.2;BP003905.1;BP002140.1;BP006936.1;BP009676.1;BP030374.1;BP034363.1;BP029458.1;BP012211.4;BP000243.5;BP033647.1;BP021195.1 | K05391+K13448+K02183+K13425+K13426+K13447+K04371+K13412+K13424+K13423+K13457+K13416+K13430+K13428+K13420+K13417+K13429+K13462 |
| RNA polymerase | 33 | 312 | 7.20E-08 | 2.40E-06 | ko03020 | BP024722.1;BP028383.1;BP004607.1;BP004397.1;BP031116.1;BP031854.1;BP008289.1;BP005223.1;BP015494.1;BP029457.1;BP037042.1;BP005978.1;BP008286.1;BP029463.1;BP008659.1;BP014705.4;BP029461.1;BP005800.1;BP014056.1;BP031855.2;BP005980.1;BP006372.1;BP015491.1;BP008288.1;BP004602.1;BP004604.1;BP027938.1;BP035218.1;BP022333.2;BP008986.1;BP016559.1;BP027935.2;BP029458.1 | K03013+K03040+K03008+K03016+K03014 |
| Pyrimidine metabolism | 37 | 458 | 8.93E-06 | 2.23E-04 | ko00240 | BP024722.1;BP028383.1;BP004607.1;BP004397.1;BP031116.1;BP031854.1;BP008289.1;BP005223.1;BP015494.1;BP029457.1;BP037042.1;BP005978.1;BP008286.1;BP029463.1;BP008659.1;BP014705.4;BP029461.1;BP005800.1;BP014056.1;BP013357.2;BP031855.2;BP005980.1;BP006372.1;BP015491.1;BP008288.1;BP004602.1;BP004604.1;BP027938.1;BP035218.1;BP018180.1;BP022333.2;BP008986.1;BP016559.1;BP027935.2;BP010316.1;BP011723.1;BP029458.1 | K03013+K03040+K03008+K03016+K03014+K00876+K14641+K01240 |
| Purine metabolism | 34 | 475 | 0.000221802 | 4.44E-03 | ko00230 | BP024722.1;BP028383.1;BP004607.1;BP004397.1;BP031116.1;BP031854.1;BP008289.1;BP005223.1;BP015494.1;BP029457.1;BP037042.1;BP005978.1;BP008286.1;BP029463.1;BP008659.1;BP014705.4;BP029461.1;BP005800.1;BP014056.1;BP031855.2;BP005980.1;BP006372.1;BP015491.1;BP008288.1;BP004602.1;BP004604.1;BP027938.1;BP035218.1;BP018180.1;BP022333.2;BP008986.1;BP016559.1;BP027935.2;BP029458.1 | K03013+K03040+K03008+K03016+K03014+K14641 |
| Glyoxylate and dicarboxylate metabolism | 8 | 73 | 0.005681171 | 8.54E-02 | ko00630 | BP022385.1;BP026136.1;BP002411.1;BP035387.1;BP035936.3;BP009900.1;BP018551.1;BP020931.1 | K02437+K11517+K00600+K01433+K15893+K01638+K15919+K01681 |
| SNARE interactions in vesicular transport | 8 | 74 | 0.006167549 | 8.54E-02 | ko04130 | BP021769.1;BP010032.1;BP006695.1;BP034048.1;BP026435.3;BP032736.1;BP033357.1;BP032737.1 | K08493+K08504+K08515+K08503+K08486+K08488 |
| Amino sugar and nucleotide sugar metabolism | 16 | 215 | 0.006830838 | 8.54E-02 | ko00520 | BP007566.1;BP030155.1;BP026207.1;BP014149.1;BP013249.1;BP026386.1;BP025773.1;BP004544.1;BP007450.1;BP026390.1;BP015178.1;BP026216.1;BP011172.1;BP019699.1;BP008072.2;BP016226.1 | K00975+K00972+K01183+K00770+K00820+K13648+K00847 |
| Vitamin B6 metabolism | 4 | 28 | 0.01922404 | 2.14E-01 | ko00750 | BP023003.1;BP023002.1;BP002272.1;BP027743.1 | K05275+K01733 |
| Linoleic acid metabolism | 3 | 24 | 0.05834483 | 5.83E-01 | ko00591 | BP010959.1;BP024089.1;BP033411.1 | K00100+K15718+K14674 |
| ABC transporters | 15 | 262 | 0.06646571 | 6.04E-01 | ko02010 | BP024812.1;BP001197.1;BP034495.3;BP021540.1;BP036883.1;BP008121.1;BP014325.1;BP032916.1;BP028486.2;BP025328.1;BP036744.1;BP013711.1;BP031422.1;BP029220.1;BP034916.1 | K05681+K05657+K05658+K05643+K05641+K05666+K05677 |
| Photosynthesis | 5 | 69 | 0.1143654 | 9.20E-01 | ko00195 | BP028382.1;BP028367.1;BP029736.1;BP010364.1;BP026585.1 | K02637+K02703+K02111+K02698+K02721 |
| Porphyrin and chlorophyll metabolism | 5 | 70 | 0.1196256 | 9.20E-01 | ko00860 | BP010959.1;BP010550.1;BP022288.2;BP002957.1;BP031019.1 | K00218+K00510+K00231+K02492+K00228 |
| Phosphatidylinositol signaling system | 6 | 94 | 0.1384675 | 9.60E-01 | ko04070 | BP028202.1;BP028215.1;BP020436.1;BP005703.2;BP003082.1;BP014236.1 | K02183+K00888+K01110 |
| Cyanoamino acid metabolism | 8 | 138 | 0.1439786 | 9.60E-01 | ko00460 | BP002411.1;BP019808.1;BP006738.1;BP027809.1;BP020533.1;BP007825.1;BP006794.1;BP007826.1 | K00600+K13030+K01501+K01188+K05350 |
| One carbon pool by folate | 2 | 24 | 0.2246748 | 1.00E+00 | ko00670 | BP002411.1;BP035387.1 | K00600+K01433 |
| beta-Alanine metabolism | 3 | 46 | 0.2449879 | 1.00E+00 | ko00410 | BP013597.1;BP010543.4;BP009672.1 | K00797+K05605+K00276 |
| Starch and sucrose metabolism | 17 | 380 | 0.2532759 | 1.00E+00 | ko00500 | BP007566.1;BP025269.1;BP013249.1;BP020474.3;BP025773.1;BP006484.1;BP020262.1;BP001122.1;BP016118.1;BP005098.1;BP007825.1;BP028310.1;BP006794.1;BP011172.1;BP024507.1;BP016226.1;BP007826.1 | K00975+K01051+K00770+K00695+K00696+K01193+K01187+K01176+K01188+K01179+K05350+K13648+K00847 |
| Propanoate metabolism | 4 | 70 | 0.2640806 | 1.00E+00 | ko00640 | BP006756.1;BP010543.4;BP026734.1;BP018351.1 | K00224+K05605+K01505+K01895 |
| Flavonoid biosynthesis | 14 | 312 | 0.2758597 | 1.00E+00 | ko00941 | BP013215.1;BP023191.1;BP027282.1;BP006756.1;BP002605.1;BP012210.1;BP012205.1;BP013297.1;BP006000.5;BP020163.1;BP024883.1;BP036002.1;BP018374.1;BP036008.1 | K13065+K05280+K13081+K09754+K08695+K13082+K13083+K00660 |
