# Supplementary material for: A Novel R2R3-MYB Transcription Factor BpMYB106 of Birch (Betula platyphylla) Confers Increased Photosynthesis and Growth Rate through Up-regulating Photosynthetic Gene Expression
Source: Front Plant Sci. 2016 Mar 22;7:315. doi: 10.3389/fpls.2016.00315 (PMC4801893; doi:10.3389/fpls.2016.00315)
Supplement: Table S5 — Analysis of MYB binding site of 10 DEGs promoters using PLACE. [file Table5.DOC]

Table S5 Analysis of MYB binding site of DEGs promoter by PLACE.

| Element name | Element position in DEGs promoters | | | | | | | | | |
| --- | --- | --- | --- | --- | --- | --- | --- | --- | --- | --- |
|  | *BP028382.1* | *BP028367.1*  *BP029736.1* | *BP010364.1* | *BP026585.1* | *BP028361.1* | *BP028375.1* | *BP028374.1* | *BP028390.1* | *BP028342.1* | *BP000067.2* |
| MYB2  (YAACKG) | -1465 (+);  -1414 (+);  -1369 (-);  -312 (-);  -35 (-) | -1595 (-);  -1546 (-) | -1070 (-);  -384 (-) | -1721 (-) | -1093 (+);  -964 (+);  -1200 (-) | -1638 (-);  -1077 (-);  -231 (-) | -20 (+);  -1595 (-);  -749 (-);  -251 (-);  -200 (-);  -124 (-) | -1381 (+);  -942 (+);  -1500 (-);  -922 (-);  -890 (-);  -85 (-) | -306 (-) | -176 (-) |
