# Supplementary material for: A Novel R2R3-MYB Transcription Factor BpMYB106 of Birch (Betula platyphylla) Confers Increased Photosynthesis and Growth Rate through Up-regulating Photosynthetic Gene Expression
Source: Front Plant Sci. 2016 Mar 22;7:315. doi: 10.3389/fpls.2016.00315 (PMC4801893; doi:10.3389/fpls.2016.00315)
Supplement: Table S6 — List of primers used for the cloning of cDNAs and plasmid constructions. [file Table6.DOC]

Table S6 List of primers used for the cloning of cDNAs or plasmid constructions.

| Primer name | Primer sequence (5’-3, the restriction enzyme sites and element sites were underlined) |
| --- | --- |
| MYB-S/A | ATGGGTCGGTCACCATGCTG / TCAAAACATTGGCGAATC |
| MYB-GFPS | CGTCGACATGGGTCGGTCACCATGCTG (*SalⅠ*) |
| MYB-GFPA | CACTAGTAAACATTGGCGAATCAGAGG (*SpeⅠ*) |
| Pro-SP1 | CTGTTCTCTTCGGCAAGTGAGTTGC |
| Pro-SP2 | TGTAGGTGATAGTGTGGAGTGAGTG |
| Pro-SP3 | CCTGTTGCCTAAGAGAGCATGGAGC |
| Pro-SP4 | TCAAACCCACCTTGTCACAGCA |
| Pro-SP5 | GAGATGTCAGCGAGATAAATGGG |
| pMYB-S | CAAGCTTGTCCTTTTAAACTATCACTTGC (*HindⅢ*) |
| pMYB-A | CTCTAGATTGGAAGTGATGATATTAGC *(XbaⅠ*) |
| p121-S/A | GCTATGACCATGATTACGCCAAGCTT / AGATCTCCTAGGGGCCCACCAGTCAG |
| MYB-OES | CGGATCCGATGGGTCGGTCACCATGCTG (*BamHⅠ*) |
| MYB-OEA | CGAGCTCGGTCAAAACATTGGCGAATC (*SacⅠ*) |
| pROK-S/A | CGCAAGACCGGCAACAGGAT / CTCCACTGACGTAAGGGAT |
