# Supplementary material for: A Novel R2R3-MYB Transcription Factor BpMYB106 of Birch (Betula platyphylla) Confers Increased Photosynthesis and Growth Rate through Up-regulating Photosynthetic Gene Expression
Source: Front Plant Sci. 2016 Mar 22;7:315. doi: 10.3389/fpls.2016.00315 (PMC4801893; doi:10.3389/fpls.2016.00315)
Supplement: Table S7 — List of primers used for the quantitative RT-PCR. [file Table7.DOC]

Table S7 List of primers used for the quantitative RT-PCR.

| Primer name | Primer sequence (5’-3’) |
| --- | --- |
| MYB-RTS/A | TCACCCACGTCAACACTTACATT / GAAGAACCTGAACTACTCCCAAC |
| 18S-RTS/A | GAGGTAGCTTCGGGCGCAACT / GCAGGTTAGCGAAATGCGATAC |
| BP028382.1-S/A | TCTGGGTGTATTGACCGCATCTT / AAACCGAGTCAGAGTGGATTGTC |
| BP028367.1-S/A | GCTTTTCTGACGGTATGCCTCTA / CATTTTCTGTGGTTTCCCTGATC |
| BP029736.1-S/A | TATGATGATCCGTCCAAACAAGC / GTAAAGCAGTCATACTTCCTTCACCTA |
| BP010364.1-S/A | AGTTCAACCAATGAGGCGTAAGG / CATGCCAAGGTATCTGCAAGAGT |
| BP026585.1-S/A | GGGTTTCAGCCTCACCTGTTCTT / CTCCGTGCTCAGTCTATCATCCA |
| BP028361.1-S/A | TCTCAGAGGCATTCTTATCATTTGG / ACGTGGATTCACGTAGAACATCG |
| BP028375.1-S/A | CGATGCTTGGCTACAATTTCGA / CCAACAATTAGGATAAGCACGA |
| BP028374.1-S/A | AGATCGAATTAGGTCTCAACAGG / CGAAGTAGATGGTGGTTGATAGA |
| BP028390.1-S/A | AGTATCGGACCATCCATAGCAGT / ACATAAGAAGTCCAATGGGAGCA |
| BP028342.1-S/A | TAGAAGGTGCAAAATCAATAGGTGC / GATCGGAATACGGATGAGATCAAAA |
| BP000067.2-S/A | CACCTCCTACATATTTCCGAACC / CGAGCTATAAGAACTAATGCTCCC |
