# Supplementary material for: A Novel R2R3-MYB Transcription Factor BpMYB106 of Birch (Betula platyphylla) Confers Increased Photosynthesis and Growth Rate through Up-regulating Photosynthetic Gene Expression
Source: Front Plant Sci. 2016 Mar 22;7:315. doi: 10.3389/fpls.2016.00315 (PMC4801893; doi:10.3389/fpls.2016.00315)
Supplement: Table S8 — List of primers used for Y1H. [file Table8.DOC]

Table S8 List of primers used for Y1H.

| Primer name | Primer sequence (5’-3’, the restriction enzyme sites and element sites were underlined) |
| --- | --- |
| ADM-S | TGGCCATTATGGCCCGGGATGGGTCGGTCACCATGCTG (*SmaⅠ*) |
| ADM-A | GACATGTTTTTTCCCGGGAAACATTGGCGAATCAGAG (*SmaⅠ*) |
| AD-S/A | GTACCCATACGACGTACCAGATTAC / ATCTACGATTCATCTGCAGCTCGAG |
| MYB2-S/A | AATTCCAACTGCAACTGCAACTGGAGCT / CCAGTTGCAGTTGCAGTTGG |
| HIS-S/A | GCCTTCGTTTATCTTGCCTGCTC / CGATCGGTGCGGGCCTCTTC |
