# Supplementary material for: A Novel R2R3-MYB Transcription Factor BpMYB106 of Birch (Betula platyphylla) Confers Increased Photosynthesis and Growth Rate through Up-regulating Photosynthetic Gene Expression
Source: Front Plant Sci. 2016 Mar 22;7:315. doi: 10.3389/fpls.2016.00315 (PMC4801893; doi:10.3389/fpls.2016.00315)
Supplement: Table S9 — List of primers used for the transient assay. [file Table9.DOC]

Table S9 List of primers used for transient assay.

| Primer name | Primer sequence (5’-3’) |
| --- | --- |
| MYB2-S | AGCTTCAACTGCAACTGCAACTGACCCTTCCTCTATATAAGGAAGTTCATTTCATTTGGAGAGAACACGGC |
| MYB2-A | CATGGCCGTGTTCTCTCCAAATGAAATGAACTTCCTTATATAGAGGAAGGGTCAGTTGCAGTTGCAGTTGA |
| p1301-S | TAGAGTCGACCTGCAGGCAT |
| p1301-A | ATCATCATCATAGACACACG |
