# Supplementary material for: A Novel R2R3-MYB Transcription Factor BpMYB106 of Birch (Betula platyphylla) Confers Increased Photosynthesis and Growth Rate through Up-regulating Photosynthetic Gene Expression
Source: Front Plant Sci. 2016 Mar 22;7:315. doi: 10.3389/fpls.2016.00315 (PMC4801893; doi:10.3389/fpls.2016.00315)
Supplement: Table S10 — Maximum quantum yield of PSII (Fv/Fm) of WT and transgenic lines. [file Table10.DOC]

Table S10 Maximum quantum yield of PSII (*Fv/Fm*) of WT and transgenic lines.

| Name of lines | *Fv/Fm* |
| --- | --- |
| WT | 0.803±0004 |
| line 1 | 0.792±0.010 |
| line 3 | 0.793±0.003 |
| line 8 | 0.806±0.008 |
| line 9 | 0.797±0.024 |
